# Sequencing, *De novo* Assembly, Functional Annotation and Analysis of *Phyllanthus amarus* Leaf Transcriptome Using the Illumina Platform

**DOI:** 10.3389/fpls.2015.01199

**Published:** 2016-01-28

**Authors:** Aparupa Bose Mazumdar, Sharmila Chattopadhyay

**Affiliations:** Organic and Medicinal Chemistry Division, Plant Biology Laboratory, Council for Scientific and Industrial Research-Indian Institute of Chemical BiologyKolkata, India

**Keywords:** *Phyllanthus amarus*, next-generation sequencing (NGS), Illumina Miseq, leaf transcriptome, *de novo* assembly, functional annotation, secondary metabolism

## Abstract

*Phyllanthus amarus* Schum. and Thonn., a widely distributed annual medicinal herb has a long history of use in the traditional system of medicine for over 2000 years. However, the lack of genomic data for *P. amarus*, a non-model organism hinders research at the molecular level. In the present study, high-throughput sequencing technology has been employed to enhance better understanding of this herb and provide comprehensive genomic information for future work. Here *P. amarus* leaf transcriptome was sequenced using the Illumina Miseq platform. We assembled 85,927 non-redundant (nr) “unitranscript” sequences with an average length of 1548 bp, from 18,060,997 raw reads. Sequence similarity analyses and annotation of these unitranscripts were performed against databases like green plants nr protein database, Gene Ontology (GO), Clusters of Orthologous Groups (COG), PlnTFDB, KEGG databases. As a result, 69,394 GO terms, 583 enzyme codes (EC), 134 KEGG maps, and 59 Transcription Factor (TF) families were generated. Functional and comparative analyses of assembled unitranscripts were also performed with the most closely related species like *Populus trichocarpa* and *Ricinus communis* using TRAPID. KEGG analysis showed that a number of assembled unitranscripts were involved in secondary metabolites, mainly phenylpropanoid, flavonoid, terpenoids, alkaloids, and lignan biosynthetic pathways that have significant medicinal attributes. Further, Fragments Per Kilobase of transcript per Million mapped reads (FPKM) values of the identified secondary metabolite pathway genes were determined and Reverse Transcription PCR (RT-PCR) of a few of these genes were performed to validate the *de novo* assembled leaf transcriptome dataset. In addition 65,273 simple sequence repeats (SSRs) were also identified. To the best of our knowledge, this is the first transcriptomic dataset of *P. amarus* till date. Our study provides the largest genetic resource that will lead to drug development and pave the way in deciphering various secondary metabolite biosynthetic pathways in *P. amarus*, especially those conferring the medicinal attributes of this potent herb.

## Introduction

*P. amarus* Schum. and Thonn., a member of family Euphorbiaceae is used in the traditional system of medicine like Ayurveda for over the centuries because of its rich medicinal values and ethnomedicinal importance. *P. amarus* Schum. and Thonn. is a small, erect annual herb whose stem has a green, smooth capsule, and grows up to 10–50 cm high. Over the last few decades, *P. amarus* has gained global recognition for its medicinal properties after several studies that were conducted to understand its therapeutic potential, yielding exciting results. However, studies about the DNA or protein sequences of this species are very limited. *P. amarus*, popularly known as “bhuiamlaki” is distributed worldwide. In Spain, this plant is best known by the common name “chanca piedra,” which means stone-breaker. The most significant hepatoprotective role of *P. amarus* has long been reported (Thyagarajan et al., 1988; Blumberg et al., 1990). The genus *Phyllanthus* has prospective beneficial therapeutic actions in the management of hepatitis B, nefrolitiase, and in painful disorders (Calixto et al., 1998). Recent studies have also reported the hepatoprotection property of *P. amarus* (Chirdchupunseree and Pramyothin, 2010; Krithika et al., 2014). In addition to this, *P. amarus* has also shown to exhibit antioxidant (Harish and Shivanandappa, 2006), antitumor and anticarcinogenic activities (Rajeshkumar et al., 2002). Besides, anti-allodynic and antioedematogenic properties, as well as antimicrobial potentiality (Mazumder et al., 2006) of this herb, have also been reported. Report of α-amylase inhibitory properties of *P. amarus* in treating diabetes has also been shown (Ali et al., 2006). The wide variety of secondary metabolites, that attribute to these medicinal properties are present mainly in the leaves and include lignans mainly phyllanthin and hypophyllanthin (Chopra et al., 1956; Rao and Bramley, 1971; Somanabandhu et al., 1993) besides nirtetralin, niranthin, diarylbutane, nyrphyllin, and a neolignan, phyllnirurin; geraniin and flavonoids like quercetin, astralgin, quercetrin, isoquercetin, and rutin (Umezawa, 2003; Kassuya et al., 2006; Leite et al., 2006). It also contains minor compounds like hydrolysable tannins like phyllanthusiin D (Foo and Wong, 1992), amariin, amarulone (Foo, 1993), amarinic acid (Foo, 1995) and alkaloids like entnorsecurinine, isobubbialine, and epibubbialine (Houghton et al., 1996).

A number of reports addressed the genetic diversity of *P. amarus* for application in the cultivar identification using PCR and sequencing based techniques viz. RFLP, RAPD, ISSR, SCAR, and AFLP (Jain et al., 2003; Senapati et al., 2011; Bandyopadhyay and Raychaudhuri, 2013). Despite its global medicinal importance genomic sequence resources available for *P. amarus* are extremely scarce. As of July 2015, only 119 ESTs, 105 genome survey sequences, and 188 nucleotide sequences are available at the National Center for Biotechnology Information (NCBI) database. Out of the 119 ESTs, 57 sequences were reported in our previous study on *P. amarus* leaves (Chattopadhyay and Bose Mazumdar Ghosh, 2014). In view of this extremely limited genome sequence, an in-depth study of transcriptome might facilitate the analysis of functional genes and thereby unravel the transcripts involved in several biological processes and mainly help understand the various metabolic pathways involved in the phytotherapeutic attributes of *P. amarus*.

The recent emergence of the next-generation sequencing (NGS) technology has made the rapid transcriptome sequencing more feasible. Previous studies have shown that development of RNA sequencing (RNA-seq) methodology has facilitated the analysis of transcriptomes of a number of models as well as non-model crop and medicinal plants (Nakasugi et al., 2013; Lehnert and Walbot, 2014; Rastogi et al., 2014). The main advantage of RNA-seq compared with the whole genome sequencing is that only transcribed regions of the genome are analyzed in the former. It is among the most popular techniques of NGS and this methodology still remains the golden standard for both coding and non-coding gene annotation. RNA-seq method offers a comprehensive and integrated view of the transcriptome revealing SNPs, novel transcribed regions, as well as the precise location of transcription boundaries (Wilhelm et al., 2010). Furthermore, RNA-seq data with NGS technologies help in assessing the process of different forms of alternative splicing from both plant and mammalian genomes as well (Rogers et al., 2012). The approach of eukaryotic transcriptome analysis is expected to get highly altered by the advanced RNA-seq technology.

In the present study, an attempt has been made to annotate and analyze the leaf transcriptome of *P. amarus* since the vast array of secondary metabolites are present substantially in the leaf tissues. We performed *de novo* transcriptome sequencing using the Illumina Miseq platform as prior genome information on *P. amarus* is unavailable. Here for RNA-seq analysis, we chose a Miseq platform because compared to other Illumina platforms the longer length of the sequencing reads generated from the Miseq platform considerably enhances the accuracy of the subsequent *de novo* assembly, besides being a rapid and cost-effective platform for transcriptome assembly and analyses. To the best of our knowledge, this is the first report of *de novo* sequencing and transcriptome analysis of *P. amarus* which will serve for the discovery of different genes involved in various metabolic pathways, especially the putative members of medicinally important secondary metabolites biosynthetic pathways and also help in the development of molecular markers like Simple Sequence Repeat (SSR) for enhancing the medicinal traits of this herb.

## Materials and methods

### Ethics statements

All necessary permits for plant sample collection for our present study were obtained. CSIR-Indian Institute of Chemical Biology, Kolkata is the authority responsible for *P. amarus* cultivation in its medicinal plant garden which provides permission to collect the samples for our scientific research.

### Sample preparation

Leaf samples of *P. amarus* Schum. and Thonn. cultivar, taxonomically identified by the Botanical Survey of India, Shibpur, Howrah, as PA202 were chosen for our study. Leaf samples from young, healthy plants were collected. RNA was extracted separately from leaf samples of the two samplings using “Roche High Pure RNA Isolation Kit,” (Product no.11828665001). The purity and concentration of each RNA sample were checked by using the Agilent 2100 bioanalyzer (Agilent Technologies, USA) before proceeding to further downstream analyses. Library preparation was performed from 1 μg of total RNA, using Illumina's “TruSeq® RNA Sample Preparation v2 Guide” (Part # 15026495 Rev. F March 2014).

### Illumina sequencing and quality control

Illumina MiSeq system was used for sequencing the *P. amarus* leaf transcriptome library using Sequencing by Synthesis (SBS) technology. The library has been sequenced following manufacturer's instructions using the MiSeq Reagent Kit v2 (Part # 15034097 Rev. B). The base-calling pipeline of Illumina's MiSeq version was MiSeq Control Software 2.2.0-RTA 1.17.28.0—CASAVA-1.8.2 which was used to generate paired-end and single-end data in FastQ format. Low-quality bases result in misassemblies by interfering in the assembly process. Hence, quality filter is the first and foremost requisite for all downstream computational analyses and results interpretation (Li et al., 2015). So additional quality control of raw data using FastQC was performed. The reads were preprocessed using Trimmomatic and SeqPrep software to obtain clean paired-end and single-end MiSeq data in a FastQ format which was also subjected to quality control using FastQC. The high quality, filtered reads were used for downstream analyses.

### *De novo* assembly and clustering

Transcriptome *de novo* assembly was carried out on three levels using both paired and unpaired high-quality reads as inputs. Velvet (version 1.2.09) and Oases (version 1.2.09) were used for first level assembly and the clean reads were split into different “k-mers” from kmer27-kmer63. The transcripts obtained from all the “kmers” were merged and assembled at level 2 using Velvet and Oases. This level 2 assembled transcript was further assembled and clustered using CD-HIT (version 4.5.4) to remove redundant transcripts (Li and Godzik, 2006). This level 3 assembly and clustering represented the final dataset of clustered non-redundant (nr) unique sequences (“unitranscripts”) for *P. amarus* leaf transcriptome.

### Gene, pathway annotation, classification

Functional annotations were performed by sequence comparison with public databases. For sequence similarity search, the annotation of unitranscripts was performed by BLASTX (Altschul et al., 1997) at NCBI using green plants (taxid: 33090) of nr protein database. The BLASTX results were imported to Blast2GO suite (Conesa and Götz, 2008) for mapping and retrieving GO terms to the assembled sequences, and further annotated with unique enzyme codes (EC) and KEGG maps (http://www.genome.jp/kegg) (Kanehisa and Goto, 2000; Kanehisa et al., 2014). GO terms are dynamic-structured, precisely defined controlled vocabulary that can be employed to describe functions of genes and gene products. These retrieved GO terms were allocated to query sequences and the extensive groups of genes present in *P. amarus* leaf transcriptome were classified into three categories - biological process, molecular function, and cellular component. Then WEGO (Ye et al., 2006) tool (http://wego.genomics.org.cn) was used to functionally classify GO terms and graphically represent the unitranscript functions at the macro level. Further BLASTX against the Clusters of Orthologous Groups database (http://www.ncbi.nlm.nih.gov/COG/) was performed to predict and classify functions of the assembled unitranscripts, using Autofact (http://megasun.bch.umontreal.ca/Software/AutoFACT.htm) tool (Koski et al., 2005).

### Comparison of *P. amarus* assembled data with closely related species

Comparison of *P. amarus* assembled unitranscripts was carried out with the most closely related species that were obtained after the functional annotation of the leaf transcriptome assembled data at NCBI green plants nr protein database. Comparison with the closely related species were performed using TRAPID analysis (Van Bel et al., 2013) with similarity search *E*-value 10e-5. *Populus trichocarpa* was the most closely related species followed by *Ricinus communis*. A comparison between both the species were performed and the latter being a member of the same spurge family, Euphorbiaceae to which *P. amarus* belongs.

### Identification of transcription factor families and its domain architecture

For identification of transcription factor (TF) families and domain mapping represented in *P. amarus* leaf transcriptome, the representative unitranscripts were enquired against the TF protein sequences at Plant TF database (PlnTFDB; http://plntfdb.bio.uni-potsdam.de/v3.0/downloads.php) by BLASTX with an *E*-value cutoff 1E-06.

### Identification of simple sequence repeats (SSRs)

SSRs were detected using MIcroSAtellite Identification Tool (MISA).

### FPKM value determination of major secondary metabolic pathway genes and reverse transcription PCR (RT-PCR) of selected secondary metabolite biosynthetic pathway genes in *P. amarus* leaf sample

For estimation of mRNA or unitranscript abundance of the major identified secondary metabolic pathway genes in the present study, FPKM values were determined. FPKM values for the unitranscripts were determined using the formula FPKM = (10^9^ × C)/(N × L), where C = Number of reads mapped to a unitranscript; N = Total mapped reads in the experiment; L = unitranscript length in base pairs. Further, RT-PCR enables the detection and identification of target mRNA transcripts. Hence, to validate our dataset, some of the assembled *P. amarus* unitranscripts that share sequence similarity to various secondary metabolite biosynthetic pathway genes and related TFs as identified revealing putative information of *P. amarus* leaf transcriptome were selected for performing RT-PCR. All primers for RT-PCR of selected secondary metabolite pathway genes were designed from the final assembled and clustered nr unique sequences (Supplementary File 1). The housekeeping gene actin was used as a control. Actin primers were designed (Acc No.: X63603) and the primer sequences were 5′-CGCGAAAAGATGACTCAAATC and 5′- AGATCCTTTCTGATATCCACG-3′. The RT-PCR products were electrophoresed on 1.2% agarose gel containing ethidium bromide.

## Results and discussions

### *De novo* assembly and clustering of *P. amarus* leaf transcriptome

**Figure 1:**
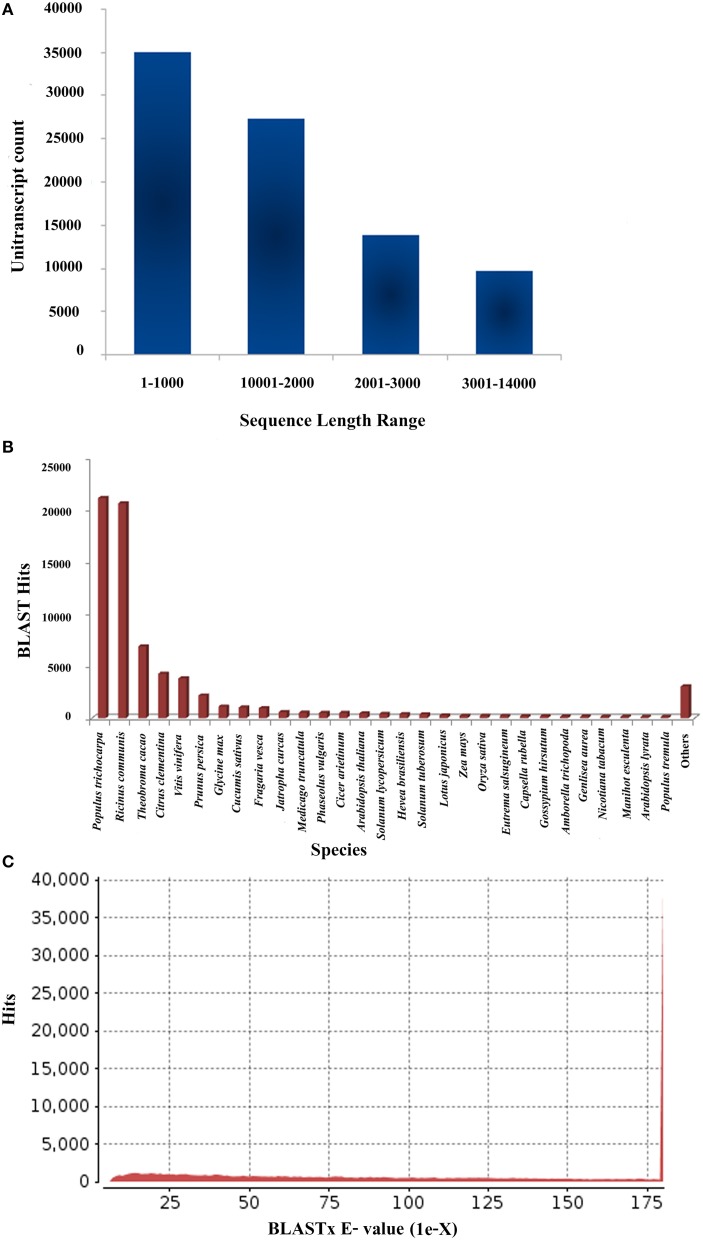
**Sequencing, *de novo* assembly, annotation of *P. amarus* leaf transcriptome. (A)** Sequence length distribution of *P. amarus* non-redundant unique unitranscript sequences. **(B)** BLASTX-Hit species distribution of *P. amarus* unitranscripts against nr protein database. **(C)** *E*-value distribution of BLAST hits against nr protein database.

The Illumina Miseq platform generated a total of 18,060,997 raw reads for *P. amarus* leaf transcriptome that accounted for approximately 9 Gbases of sequence data. The raw data was also deposited in the National Center for Biotechnology Information's (NCBI) Short Read Archive (SRA) database under the accession number PRJNA248079. Raw reads were further subjected to quality control (FastQC). After quality and adaptor trimming using Trimmomatic 0.30 tool, 14,608,389 high quality paired reads, 2,291,081 (unpaired Reads R1) and 371,454 (unpaired Reads R2) were retained. All these filtered paired and unpaired reads were used in the transcriptome assembly. The summary of the filtration of total raw reads generated after RNA-seq and used further for transcriptome assembly is illustrated in Supplementary Figure 1. Filtered paired and unpaired reads were assembled using Velvet (version 1.2.09) and Oases (version 1.2.09) and the clean reads obtained were split into different k-mers from kmer27-kmer63. As a function of k-mer various output parameters were analyzed in our level 1 assembly. These parameters included—total number of transcripts, transcripts with length 100 bp and above, N50 length, longest transcript length, and average transcript length. The number of clean transcripts obtained in each kmer along with its total length, the average size and N50 value are summarized in Supplementary Table S1. For accuracy in *P. amarus* leaf transcriptome *de novo* assembly, we assembled and further merged the transcripts from kmer27-kmer63 using Velvet and Oases with long read option to obtain 360,405 transcripts in level 2 single merged assembly. To remove redundancy of the merged assembled transcripts, we used CD-HIT (version 4.5.4) to merge the level 2 assembled sequences further. Merging of the assembled transcripts resulted in 85,927 unitranscripts with maximum and minimum read lengths being 13,600 and 200 bp respectively, with an average size of the assembled unitranscripts being 1548 bp which indicated an increased coverage as well as the depth of our sequencing data. The outline of the level 2 and 3 assemblies has been summarized in Supplementary Table S2. The sequence length along with BLASTX hit and *e*-value distribution of *P. amarus* unitranscripts have been shown in Figure 1.

### Similarity search and functional annotation of *P. amarus* leaf transcriptome

**Figure 2:**
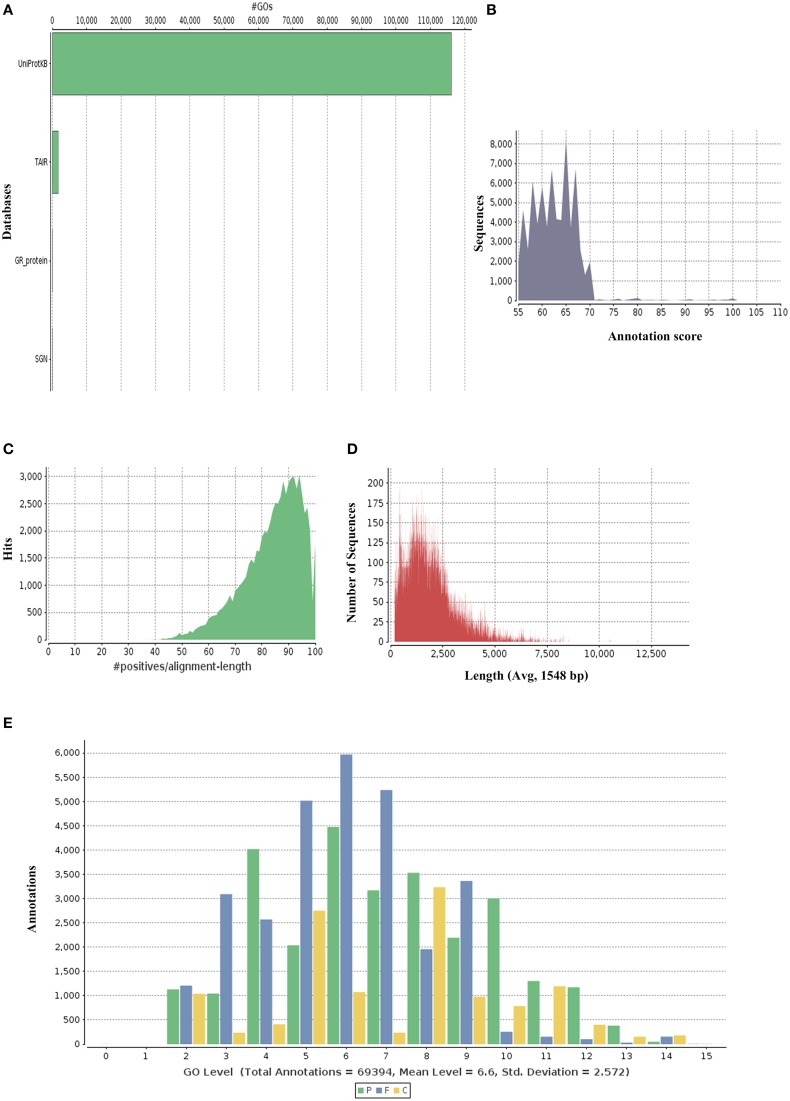
**Graphical representations of functional annotations in *P. amarus* leaf transcriptome. (A)** Representation of mapping databases (UniprotKB, TAIR) sources. **(B)** Annotation score distribution of assembled unitranscripts. **(C)** Sequence similarity distribution graph. **(D)** Distribution of annotated sequences with length. **(E)** GO level distribution of annotated unitranscript sequences.

*P. amarus* being a non-model plant of medicinal value without any prior genome information, sequence similarity search and comparison for the assembled unitranscripts of *P. amarus* leaf transcriptome was carried out by BLASTX against the green plants of nr protein database at NCBI, with an *E*-value cut off 1E-06 (Supplementary File 2). The BLASTX hit results showed that about 60.58% of the annotated descriptions were uninformative (e.g., “unknown,” “unnamed,” “putative,” or “hypothetical” protein) as a result of inadequate *P. amarus* genome information. Our dataset showed that the percentage of uninformative BLASTX hit results were nearly similar to endangered medicinal plant species *Chlorophytum borivilianum* (Kalra et al., 2013). Also, 15,265 out of 85,927, i.e., 17.76% unitranscripts were without any hits in plant nr database. In our dataset unitranscripts showed significant similarity to *Populus trichocarpa*, followed by *Ricinus communis, Theobroma cacao* and so forth (Figure 1B). BLAST2GO suite was then used for functional annotation using the BLASTX results. Graphical representation of different levels of annotations of *P. amarus* unitranscripts by BLAST2GO showing mapping against different databases (UniprotKB, TAIR, etc.), annotation score distribution of assembled unitranscripts, sequence similarity distribution, distribution of annotated sequences with length, GO level distribution of annotated unitranscript sequences have been shown in Figure 2. Blast2GO is a suitable tool for plant genomics research, especially for the large-scale functional annotation and data mining of novel sequence data of non-model species. BLASTX results were used for mapping to retrieve GO terms and further annotate to retrieve the EC (EC number). To reveal molecular interaction network and metabolic pathways, KEGG pathway annotation for the assembled unitranscripts of *P. amarus* leaf transcriptome was performed by mapping the sequences obtained from BLAST2GO to the contents of the KEGG metabolic pathway database. Annotation summary of the assembled *P. amarus* unitranscripts has been specified in Supplementary Table S3.

### Sequence similarity and comparison of *P. amarus* data with related species

Functional annotation of the assembled unitranscripts of *P. amarus* leaf transcriptome at NCBI green plants nr protein database showed high similarity to *Populus trichocarpa* and *Ricinus communis*. So comparison of the assembled data was performed with both these species. Both the species are known to possess medicinal values like anti-inflammatory, analgesic being common to both besides other properties and also *R. communis* belong to the same spurge family, Euphorbiaceae like *P. amarus*, as already mentioned. So we aimed to compare our sequenced assembled data with both the species using TRAPID analysis, which aids in the generation of detailed gene catalogs, especially for non-model species. Out of the total 85,927 unitranscripts, 71,896 (83.7%) unitranscripts showed similarity to *P*. *trichocarpa* while that of 71,358 (83%) unitranscripts showed similarity to *R*. *communis*. The detailed comparison of *P. amarus* leaf transcriptome data with both the species showing the Meta annotation, Gene Family and Functional Annotation information have been shown in Supplementary Table S4.

### Functional classifications by gene ontology (GO)

The GO database is a significant web resource in the bioinformatics field. GO provides a set of dynamic, controlled and structured vocabularies for describing the roles of genes and their products in any organism (Ashburner et al., 2000). The three categories of the GO database are—biological process, molecular function and cellular component. *P. amarus* unitranscripts with nr annotations were functionally annotated with “GO terms” by BLAST2GO suite. Further WEGO software was used for the GO functional classification of the assembled *P. amarus* unitranscripts at the macro level.

A total of 20,582 *P. amarus* unitranscripts were assigned to 69,394 GO terms and one unitranscript was assigned more than one GO term. The majority of GO terms was assigned to molecular function (29,125, 41.97%), followed by biological process (27,577, 39.74%) and cellular component (12,692, 18.29%) was the least.

**Figure 3:**
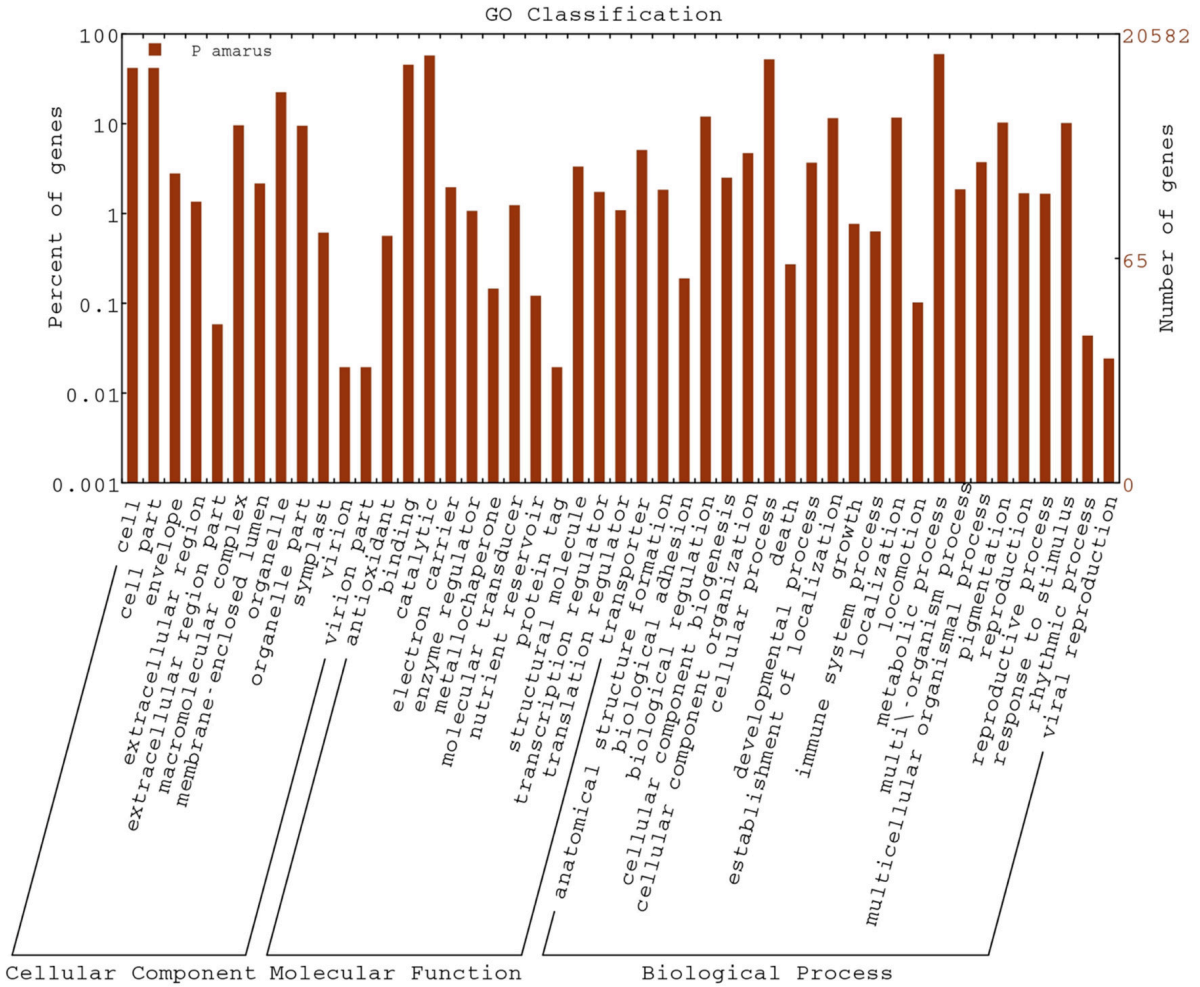
**GO functional classifications using WEGO**.

Regarding the cellular component ontology, “cell” (GO: 0005623), “cell part” (GO: 0044464), and “organelle” (GO: 0043226) were the most representative category. Under molecular function ontology, results showed a high percentage of genes from “binding” (GO: 0005488) and “catalytic activity” (GO: 0003824). Some percentage of genes were also involved in “antioxidant activity” (GO: 0016209) in the molecular function ontology as well. Moreover, biological process ontology contained mainly genes involved in “metabolic process” (GO: 0008152), “cellular process” (GO: 0009987). Figure 3 shows the categorization of *P. amarus* unitranscripts into three main ontologies and 47 sub-groups.

### Functional classifications by COG

**Figure 4:**
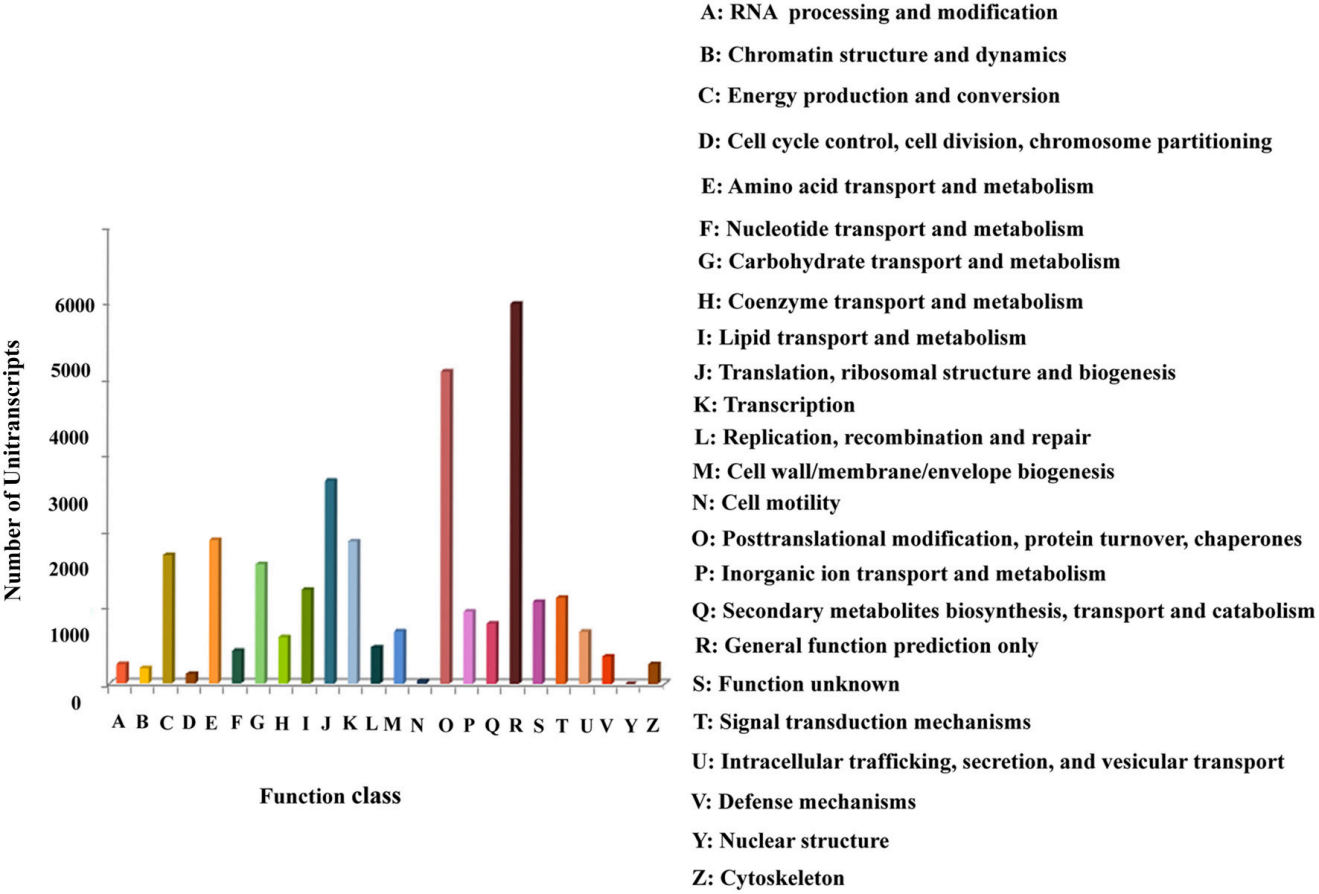
**Clusters of Orthologous Groups (COG) classification of *P. amarus* unitranscripts**.

The Cluster of Orthologous Groups (COG) database classifies orthologous gene products. The unitranscripts obtained in our study were aligned to the COG database to predict and classify their possible functions (Supplementary File 3). COG classification of the assembled unitranscripts showed that 28,121 (32.7%) unitranscripts were clustered into 24 functional categories (Figure 4). Among the different COG classes, the highest number of unitranscripts were clustered into the “general function prediction only” category (5003, 17.791%) followed by “posttranslational modification, protein turnover, chaperones” (4110, 14.615%), “translation, ribosomal structure and biogenesis” (2671, 9.498%), “amino acid transport and metabolism” (1889, 6.717%), “transcription” (1872, 6.657%), “energy production and conversion” (1687, 6.00%), “carbohydrate transport and metabolism” (1569, 5.579%), “lipid transport and metabolism” (1234, 4.388%), “signal transduction mechanisms” (1136, 4.04%). Only a few unitranscripts were assigned to “cytoskeleton,” “chromatin structure and dynamics,” “cell motility,” and “nuclear structure” (261, 199, 35, and 2, respectively). Also, 950 and 431 unitranscripts were clustered into “inorganic ion transport and metabolism,” and “nucleotide transport and metabolism” respectively. Interestingly our dataset also showed that 792 unitranscripts that constituted 2.8% of total unitranscripts annotated with COG database represented the “secondary metabolites biosynthesis, transport and catabolism” category which indicates the large number of secondary metabolites present in *P. amarus*. This finding is very similar to the previous results of KOG classification studied in another rhizomatous perennial plant *Curcuma longa* with significant therapeutic potentials (Annadurai et al., 2013).

### Pathway analysis by KEGG

**Figure 5:**
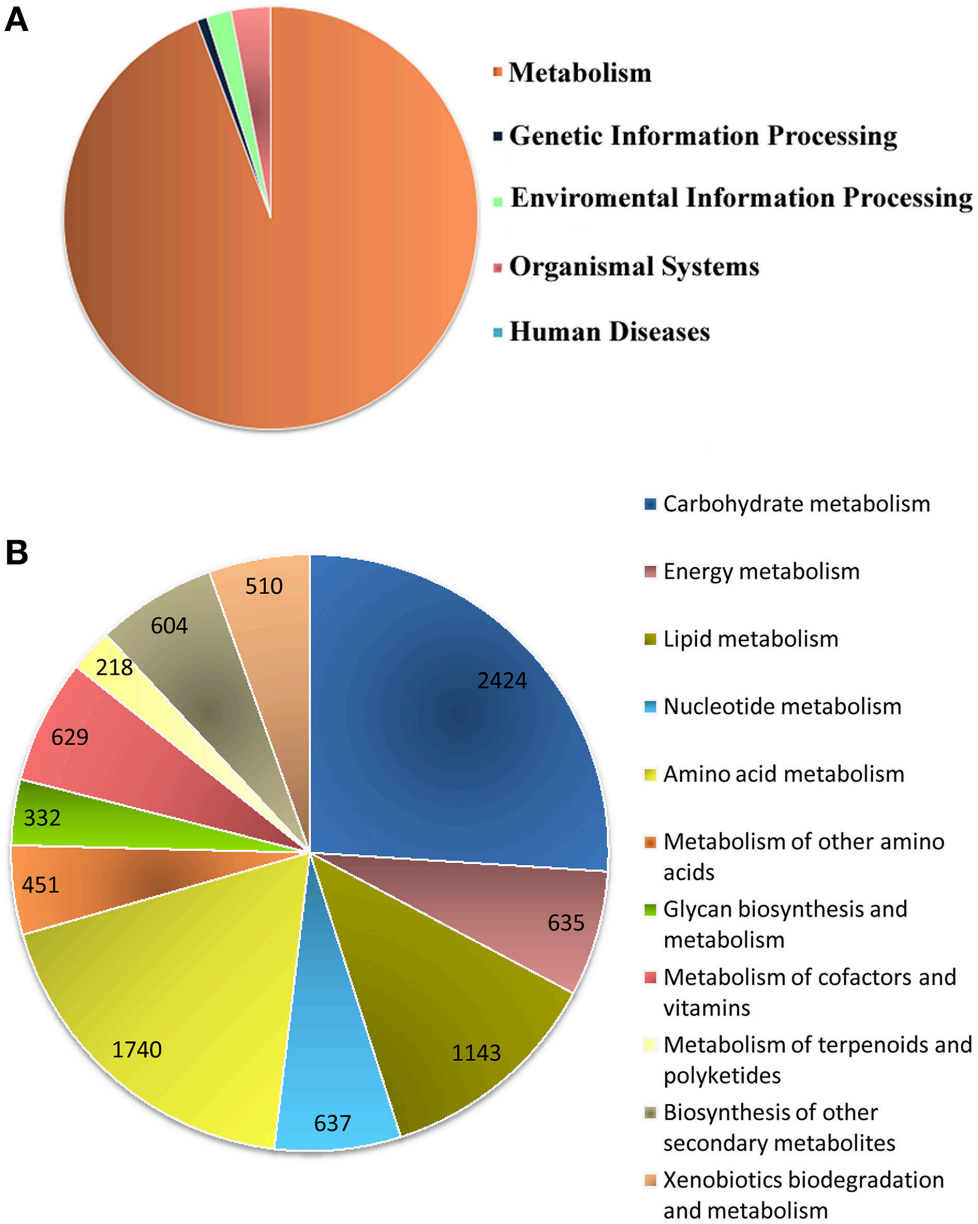
**Annotation of *P. amarus* unitranscripts by KEGG database. (A)** Distribution of *P. amarus* unitranscripts into KEGG biological categories. **(B)** Classification of *P. amarus* leaf transcriptome into KEGG “Metabolism” category.

Biological pathway studies play a key role in gaining insight into the advanced studies of genomics. KEGG is a highly integrated database providing information of the biological systems and their relationships at the molecular, cellular and organism levels, particularly via the KEGG pathway maps (Kanehisa et al., 2008). KEGG pathway annotations and EC were generated (Supplementary File 4) from the assembled unitranscripts of *P. amarus* leaf transcriptome that were mapped with GO terms. In total, 4,697 *P. amarus* unitranscripts were assigned to 134 KEGG maps and 583 EC and these EC were then used to retrieve and color the KEGG pathway maps to represent the putatively identified genes involved in several metabolic pathways. Interestingly, in our dataset it was seen that more than one unique sequence of *P. amarus* leaf transcriptome was annotated as the same enzyme. Enzymes encoded by annotated unitranscripts were grouped into the 5 major pathways in the KEGG pathway database (Figure 5A)—“metabolism” (9323 unitranscripts), “genetic information processing” (78), “environmental information processing” (191), “organismal systems” (303), “human diseases” (2). Apparently “metabolism” being one of the most significant and the most highly represented category in our study led to the in-depth analysis of this and has been represented in Figure 5B. In our dataset, it was seen that the maximum number of unitranscripts fell under the “carbohydrate metabolism” (2424 unitranscripts) followed by “amino acid metabolism” (1740 unitranscripts), “lipid metabolism” (1143 unitranscripts), “nucleotide metabolism” (637 unitranscripts). Nucleotide metabolism plays a vital role in plants for metabolism and development like other organisms. Besides, although lipid metabolism closely relates to oil plants mostly, and since *P. amarus* is a plant of therapeutic importance, lipid metabolism is also associated with plants in general (Mazliak, 1973). A number of *P. amarus* unique sequences (1332 unitranscripts) were involved in secondary metabolism as well (Supplementary Table S5). Out of all secondary metabolite pathways, “flavonoid biosynthesis” pathway was shown to be encoded by the highest number of *P. amarus* assembled unitranscripts (134 unitranscripts) followed by “phenylpropanoid biosynthesis” (125 unitranscripts). The entire functional KEGG pathway categorization of *P. amarus* leaf transcriptome unitranscripts have been shown in Supplementary Table S6.

### Analysis of secondary metabolic pathway genes

#### Lignan biosynthetic genes

**Figure 6:**
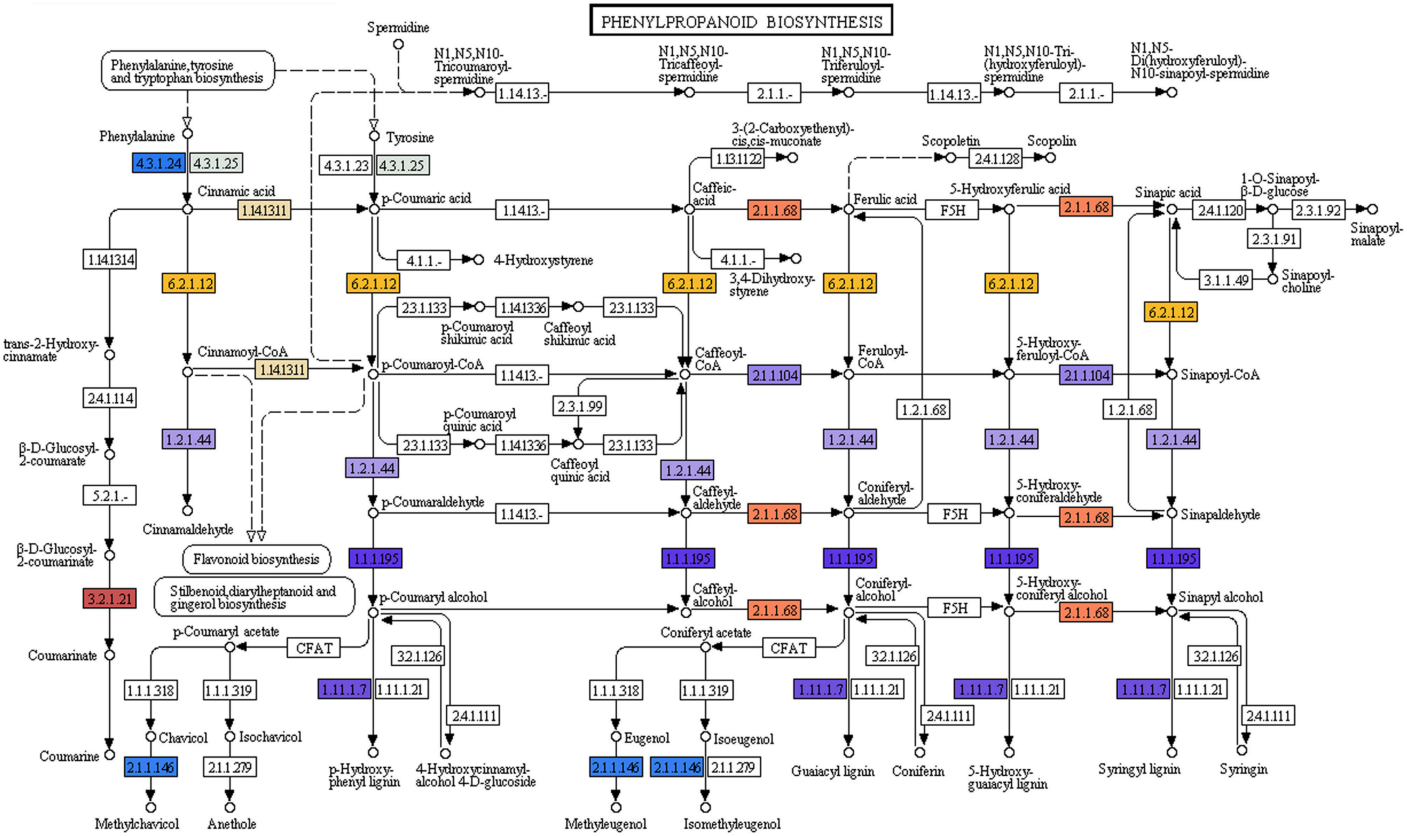
**Phenylpropanoid biosynthesis pathway study by KEGG analysis showing the different identified enzymes (one color for each Enzyme Code or EC)**.

Phenylpropanoids which comprise a large group of plant-based natural compounds is derived from phenylalanine (Michal, 1999). Phenylpropanoid biosynthesis pathway starts with the formation of cinnamic acid from phenylalanine, which leads the formation of cinnamoyl-CoA, *p*-coumaroyl-CoA, feruloyl-CoA, and sinapoyl-CoA. These CoA-activated compounds are starting metabolites for the synthesis of lignans, flavonoids, flavones, and flavonols, etc. In the present study KEGG analyses of *P. amarus* leaf transcriptome sequences revealed the presence of 11 genes involved in the biosynthesis of different compounds of phenylpropanoid pathway (Figure 6).

The major lignans reported in *P. amarus*—phyllanthin and hypophyllanthin are known to possess significant hepatoprotective and antioxidant properties. But the exact biosynthetic pathway leading to the formation of phyllanthin and hypophyllanthin is still under investigation. The structural similarity between the skeleton of secoisolariciresinol and phyllanthin is suggestive of the derivation of phyllanthin/hypophyllanthin from secoisolariciresinol. The presence of pinoresinol/lariciresinol reductase gene was indicated due to the presence of (+) secoisolariciresinol in species of *Phyllanthus* (Umezawa et al., 1997). In our leaf transcriptomic data, one unique sequence (unitranscript 77577) was assigned the pinoresinol reductase activity gene ontology term (GO: 0010283) after mapping the assembled *P. amarus* unitranscripts into BLAST2GO suite. Another six unique sequences (unitranscripts 63241; 63242; 63243; 63244; 63245; and 63246) encoding phenylcoumaran benzylic ether reductase (PCBER) - like protein were annotated the lignan biosynthetic process GO term (GO: 0009807). Besides, PCBER has also shown to have high sequence similarity with PLR, i.e., pinoresinol/lariciresinol reductase (Gang et al., 1999; Vander Mijnsbrugge et al., 2000), showing the active involvement of *P. amarus* leaves in lignans biosynthesis and thus complementing its phytotherapeutic properties.

#### Study of flavonoid biosynthesis and related pathway genes in the *P. amarus* leaf transcriptome

**Figure 7:**
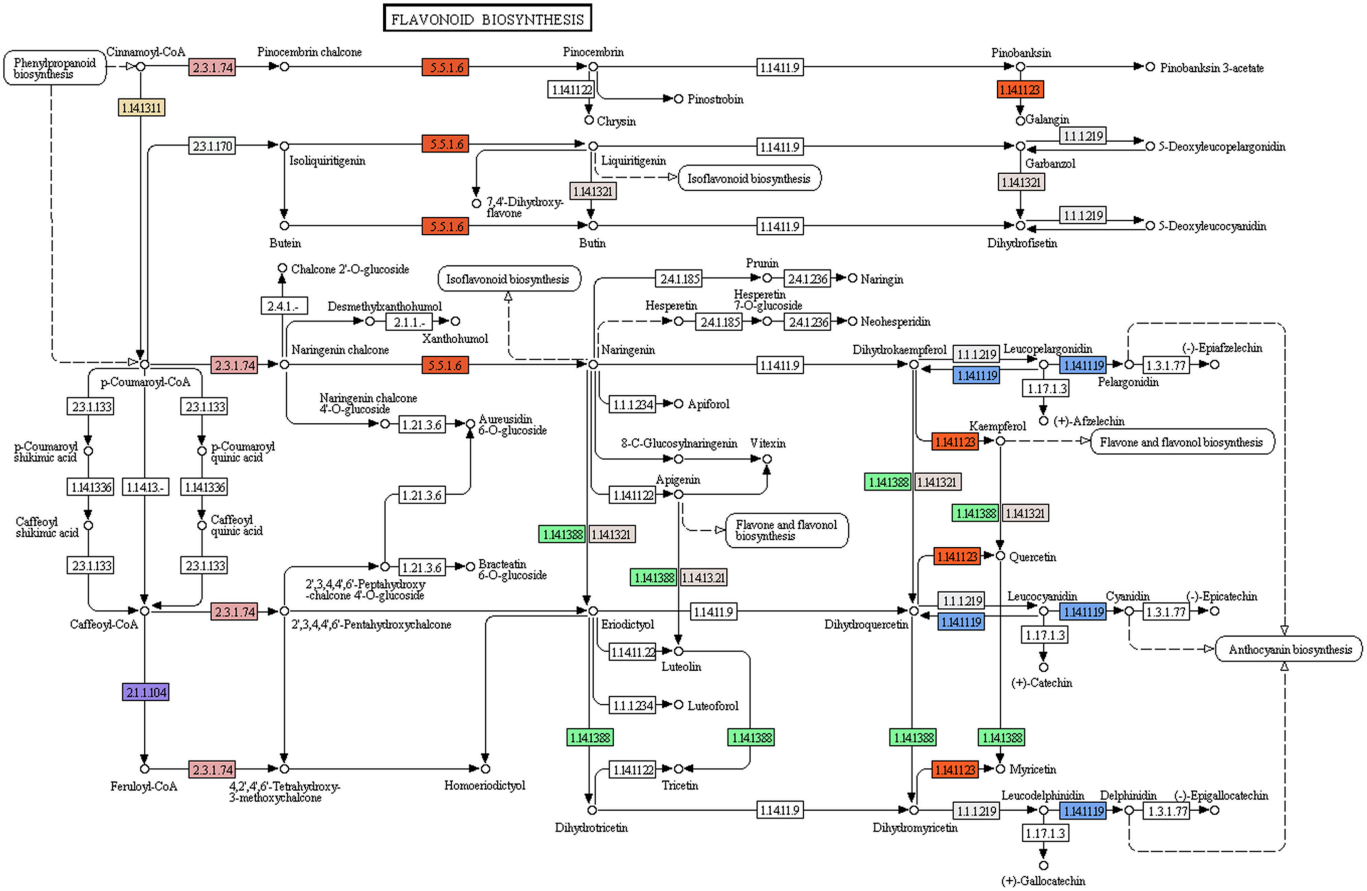
**Flavonoid biosynthesis pathway study in *P. amarus* leaf transcriptome representing each of the identified colored EC**.

Flavonoids, a class of plant secondary metabolites, are polyphenolic compounds that are categorized into flavanone, flavones, flavonols, isoflavones, catechins, chalcones and their derivatives. Due to the diverse beneficial effects of flavonoids, we chose to study the flavonoid biosynthesis and related pathway genes that were detected in the present study (Figure 7). In this dataset, starting from initial enzymes of flavonoids biosynthesis (via the phenylpropanoid pathway) like phenylalanine ammonia lyase (EC: 4.3.1.24, EC: 4.3.1.25), cinnamate 4- monooxygenase (EC: 1.14.13.11), 4-coumarate CoA ligase (EC: 6.2.1.12), and chalcone synthase (EC: 2.3.1.74) were identified. Besides, chalcone isomerase (EC: 5.5.1.6) that catalyzes chalcone isomerisation into naringenin was also found in the present study. Further, the enzymes required for naringenin conversion to produce eriodictyol and dihydrotricetin by flavonoid 3′- monooxygenase (EC: 1.14.13.21) and flavonoid 3′, 5′-hydroxylase (EC: 1.14.13.88) respectively were also identified. In addition to these, the enzyme 6′-deoxychalcone synthase (EC: 2.3.1.170) required to convert p-Coumaroyl CoA to isoliquiritigenin to produce the flavonoid butein was also found. The enzyme chalcone isomerase (EC: 5.5.1.6) also helps butein further produce another flavonoid butin. Moreover, *P. amarus* leaf transcriptome dataset also contained enzymes like flavonol synthase (EC: 1.14.11.23) and leucoanthocyanidin dioxygenase (EC: 1.14.11.19). A similar set of genes of the flavonoid biosynthesis has been reported in the leaf tissues of endangered medicinal herb *Chlorophytum* (Kalra et al., 2013). These putative findings support our present dataset in showing how the flavonoids biosynthesis pathway genes complement the therapeutic significance of *P. amarus*. The KEGG pathway analysis showed that the genes reported in the present study were required for the synthesis of several other flavonoids like pinostrobin, butein, naringenin, galangin, butin, garbanzol, dihydrofisetin (futin), eriodictyol, homoeriodictyol as well as flavones and flavonols like kaempferol, astragalin, quercetin, myricetin, and luteolin. These flavonoids (including flavones and flavonols) are known to exhibit hepatoprotective, antioxidant, anti-inflammatory, antimutagenic, antiviral, etc. properties that support these reported phytotherapeutic potentials of *P. amarus* as well. Some of the various reported bioactive effects of these flavonoids on human health are summarized below.

For instance, pinostrobin has been shown to possess chemopreventive and antioxidant properties (Fahey and Stephenson, 2002), antiviral effect (Wu et al., 2011). The flavonoid naringenin has been shown to possess hepatoprotective and antioxidant effects (Hermenean et al., 2014), anti-inflammatory as well as anticancer activities showing its preventive measures in oral carcinogenesis, hepatocarcinogenesis, and colorectal cancer (Arul and Subramanian, 2013; Sulfikkarali et al., 2013; Li et al., 2014). Recent reports show naringenin to possess neuroprotective effect against Parkinson's disease-related pathology (Lou et al., 2014), iron-induced neurotoxicity and protection of ocular ischemic diseases (Kara et al., 2014). Garbanzol has been shown as an antimutagenic flavonoid (Park et al., 2004). The flavanonol dihydrofisetin (also known as fustin), a type of flavonoid showed protective effects on neuronal cell death (Park et al., 2007). Galangin, another bioflavonoid suggested as a potential candidate for further development of new drugs against Alzheimer's disease (Guo et al., 2010) also has anticancer (Zhang et al., 2013) and hepatoprotective properties (Wang et al., 2013). Butin inhibit aromatase in the human body (Park et al., 2014). Butein suppresses breast and lung cancer (Cho et al., 2014; Seo and Jeong, 2014). Another important flavonoid reported in *P. amarus* is quercetin, widely accepted as a potent antioxidant also shows anticarcinogenic and hepatoprotective (Ji et al., 2014) activities. Both quercetin and myricetin possess antimicrobial properties (Rashed et al., 2014). Further the well-recognized flavonoid of *P. amarus*, viz. kaempferol has antioxidant, hepatoprotective and anticancer effects (Huang et al., 2014; Shakya et al., 2014; Dang et al., 2015). The flavone luteolin too has anticancer potential (Lim do et al., 2007). Recent studies have also reported that luteolin along with quercetin reduces high blood cholesterol levels *in vivo* (Nekohashi et al., 2014). Astragalin is another flavone known to possess antioxidant effects as well (Choi et al., 2013). The pathway genes of all these flavonoids were reported in our putative leaf transcriptome dataset of *P. amarus*.

#### Terpenoids and alkaloid biosynthesis pathway genes

**Figure 8:**
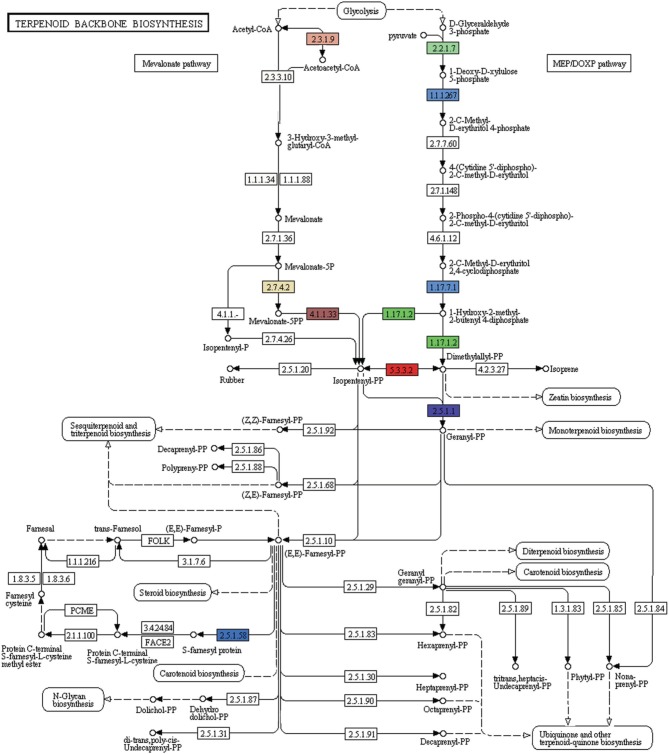
**KEGG analysis showing genes involved in MVA, MEP pathways forming the terpenoid backbone biosynthesis (Each EC with one color)**.

A number of terpenoids and alkaloid metabolism related genes were also revealed in *P. amarus* leaf transcriptome dataset. Like lignans, flavonoids, and other polyphenolic compounds, terpenoids have also shown to possess therapeutic effects in many clinical studies. Terpenoids are derived from geranyl pyrophosphate (GPP). GPP is synthesized via the cross talks between the cytosolic mevalonate (MVA) pathway and plastidial 2-C-methyl-D-erythritol-4-phosphate (MEP) or DOXP pathway products viz. isopentenyl pyrophosphate (IPP) and dimethylallyl pyrophosphate (DMAPP). MVA pathway starts with the formation of acetoacetyl-CoA while MEP/DOXP pathway starts with D-glyceraldehyde 3-phosphate (Eisenreich et al., 1998). Both MVA and MEP pathways are part of the terpenoid backbone biosynthesis (Figure 8). In the present study, multiple transcripts encoding some of the known enzymes involved in the MVA pathway, MEP/DOXP pathway, and the terpenoids biosynthesis pathway were identified. Genes involved in MVA pathway that were found in our leaf transcriptome included acetyl-CoA acetyltransferase (EC: 2.3.1.9), HMG-CoA synthase (EC: 2.3.3.10), HMG-CoA reductase (EC: 1.1.1.34), phosphomevalonate kinase (EC: 2.7.4.2), mevalonate diphosphate decarboxylase (EC: 4.1.1.33). MEP/DOXP pathway genes included 1-deoxy-D-xylulose-5-phosphate synthase (EC: 2.2.1.7), 1-deoxy-D-xylulose-5-phosphate reductoisomerase (EC: 1.1.1.267), 4-hydroxy-3-methylbut-2-enyl diphosphate synthase (EC: 1.17.7.1), 4-hydroxy-3-methylbut-2-enyl diphosphate reductase (EC: 1.17.1.2), isopentenyl-diphosphate delta isomerase (EC: 5.3.3.2). Interestingly, in the terpenoid backbone biosynthesis pathway farnesyltransferase (EC: 2.5.1.58) gene was also found to be present in putative *P. amarus* leaf transcriptome dataset. The enzyme farnesyltransferase has been one of the most attractive and fascinating targets in cancer research over the past few decades in the development of anticancer drugs (Sousa et al., 2008). Squalene synthase (EC: 2.5.1.21) and squalene monooxygenase (EC: 1.14.13.132)—the two enzymes involved in the synthesis of terpenoids precursors viz. squalene and squalene-2, 3-epoxide (Supplementary Figure 2) were also identified in the present dataset.

**Figure 9:**
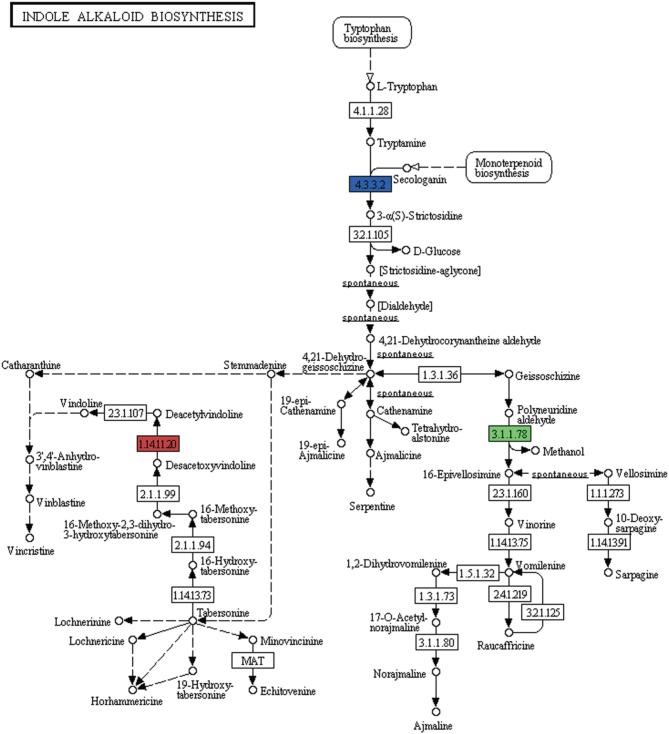
**Indole alkaloid biosynthetic pathway genes found in *P. amarus* leaf transcriptome are depicted by the different colored ECs (one color for each EC)**.

Alkaloids are a large class of naturally occurring organic nitrogen-containing chemical compounds found primarily in plants (Robinson, 1974). Several plant-based alkaloids like morphine, piperine, caffeine, quinine, strychnine, brucine, vinblastine, vincristine colchicine, etc. and their uses have long been reported (Kutchan, 1995). In *P. amarus* leaf transcriptome dataset, in addition to flavonoids (including flavones and flavonols) and terpenoids, few genes were involved in indole alkaloid (Figure 9), isoquinoline (Supplementary Figure 3) as well as tropane, piperidine and pyridine alkaloid (Supplementary Figure 4) biosynthesis pathways. Polyneuridine-aldehyde esterase (EC: 3.1.1.78), strictosidine synthase (EC: 4.3.3.2), deacetoxyvindoline 4-hydroxylase (EC: 1.14.11.20) were the genes involved in the indole alkaloid biosynthetic pathway. Unique sequences encoding for enzymes like polyphenol oxidase (EC: 1.10.3.1), primary amine oxidase (EC: 1.4.3.21), N-methylcoclaurine 3′-monooxygenase (EC: 1.14.13.71) and reticuline oxidase (EC: 1.21.3.3) in the isoquinoline alkaloid biosynthesis pathway were also revealed. Tropinone reductase I (EC: 1.1.1.206) which synthesize tropine, a derivative of tropane was also present in our dataset.

*P. amarus* leaf transcriptome dataset also revealed some antimicrobial-related pathways like—“penicillin and cephalosporin biosynthesis,” “tetracycline biosynthesis,” “polyketide sugar unit biosynthesis,” “stilbenoid, diarylheptanoid and gingerol biosynthesis,” “biosynthesis of ansamycins,” “butirosin and neomycin biosynthesis,” “streptomycin biosynthesis,” “novobiocin biosynthesis.”

*P. amarus* possesses anticancer properties as we have already discussed, owing to the diverse pharmacological properties of the different polyphenolic compounds. Interestingly, in our present study, we reported the presence of some of the unique sequences coding for enzymes having similarity with the genes involved in taxol biosynthesis. KEGG analysis showed the presence of enzymes O-acetyltransferase (EC: 2.3.1.162), 13-alpha-hydroxylase (EC: 1.14.13.77), III-10-O-acetyltransferase (EC: 2.3.1.167) that showed similarity with the genes involved in taxol biosynthesis in diterpenoid pathway (Supplementary Figure 5). Few sequences coding enzymes like dehydrogenase (EC: 1.1.1.207 and EC: 1.1.1.208) having similarities with genes involved in the menthol biosynthesis pathway were also reported in this study in the monoterpenoid biosynthesis pathway of the KEGG database. Previous study shows that menthol has a potent anticancer property (Lu et al., 2007). A summary of a few of the putatively identified major genes involved in phenylpropanoid and flavonoids as well as terpenoids and alkaloid biosynthesis pathways has been represented in Tables 1, 2, respectively.

**Table 1:** **Summary of few major genes involved in Phenylpropanoid and Flavonoid biosynthesis pathways identified putatively from *P. amarus* leaf transcriptome**.

**Gene name**	**EC number**	**Unitranscript ID**	**Unitranscript length**	**BLASTX *E*-value**	**Accession Version**	**Total unitranscripts**
Phenylalanine Ammonia Lyase (PAL)	4.3.1.24 4.3.1.25	Unitranscript 1386, 31401,31403, 31404,31405, 31408,31409, 31410,31411, 31412	2379, 2553, 1162, 1643, 1186,2574, 2541,2528, 2725,2521	0,0,0,0,0,0,0,0,0,0	XP_002519521.1, XP_002531677.1	10
Cinnamate 4-hydroxylase/ trans-cinnamate 4-monooxygenase	1.14.13.11	Unitranscript 31571, 79728, 79729	1331, 622, 610	0, 2.53E-095, 8.08E-094	XP_002523952.1 XP_002331408.1	3
Flavonoid 3′- monooxygenase	1.14.13.21	Unitranscript 67104, 74675	1154, 1948	2.93E-110, 0	XP_002533334.1 XP_002531093.1	2
Flavonoid 3′, 5′-hydroxylase	1.14.13.88	Unitranscript 2751, 29623, 48352,48353, 48354,48355, 48356,48357, 48358,48359,48360 48361,48362,48363, 48364,48365,48366, 48367, 48370,48371, 48373,57668,75534, 78374	5660,1912, 876,1272, 1836,2106, 325,5157, 3543,4142, 3656,3657, 5270,4037, 3582,5044, 5045,2296, 1430,3024, 3599,1475, 340,205	0,0, 1.05E-053,9.11E-117,0,0, 4.15E-041, 0,0,0,0,0,0,0,0,0,0,0, 2.54E-087, 0,0, 6.90E-147, 5.66E-016,2.78E-032	XP_002528647.1 XP_002510313.1 XP_002531094.1 XP_002509592.1	24
Chalcone synthase	2.3.1.74	Unitranscript 12832, 12833,12834, 12836	5097, 3769, 4959,6345	0,0,0,0	XP_002529257.1	4
Chalcone isomerase	5.5.1.6	Unitranscript 5340, 5341,42537, 42539,42540, 71988,71989	863,960,838,1322,1210, 637,908	2.74E-104, 1.12E-105, 1.35337E-13, 9.98106E-13, 7.98495E-13, 1.14E-078, 4.48E-111	XP_002315258.1	7
Flavonol synthase	1.14.11.23	Unitranscript 29419,33161, 77019,77020	1822,383, 1448,1543	6.76E-089, 4.87E-036,0,0	XP_002531459.1 XP_002533264.1 XP_002519769.1	4
Leucoanthocyanidin dioxygenase	1.14.11.19	Unitranscript 13933, 13934,13942, 13943,70616, 70617	515,512,398,466,1445, 1484	2.64E-033 2.92E-032 2.95E-027 3.53E-027 1.48E-166 2.34E-166	XP_002533635.1 XP_002522603.1	6

**Table 2:** **Summary of few major genes involved in Terpenoid and Alkaloid biosynthesis pathways identified putatively from *P. amarus* leaf transcriptome**.

**Gene name**	**EC number**	**Unitranscript ID**	**Unitranscript length**	**BLASTX *E*-value**	**Accession Version**	**Total unitranscripts**
HMG-CoA synthase	2.3.3.10	Unitranscript 62630	1906	0	XP_002509692.1	1
HMG-CoA reductase	1.1.1.34	Unitranscript 1329,30497, 30499,30500, 30501,30502, 30503,30504, 30505,30506, 30507,30508, 30509,30510	4686,973, 747,744, 3279,3222, 3064,3041, 2475,2531, 4548,3894, 3951,4476	0, 6.75E-126, 1.93E-062, 1.76E-127, 0,0,0,0,0,0,0, 0,0,0	XP_002510732.1	14
Mevalonate diphosphate decarboxylase	4.1.1.33	Unitranscript 2488, 45660	3562, 1734	0,0	XP_002521172.1	2
1-deoxy-D-xylulose-5-phosphate synthase	2.2.1.7	Unitranscript 5233,6343, 45230, 45231,53517, 53519,53520, 53521,53523, 53524,71232, 80377	2774, 1120, 2604,2671, 2770, 2618, 623,2821, 2639,2704, 844, 655	0,0,0,0,0, 0, 2.92E-027,0, 0,0, 9.55E-148, 1.46E-089	XP_002514364.1 XP_002533688.1	12
1-deoxy-D-xylulose-5-phosphate reductoisomerase	1.1.1.267	Unitranscript 54720, 54721	2158, 2108	0,0	XP_002511399.1	2
Mevalonate diphosphate decarboxylase	4.1.1.33	Unitranscript 2488, 45660	3562, 1734	0, 0	XP_002521172.1	2
4-hydroxy-3-methylbut-2-enyl diphosphate reductase	1.17.1.2	Unitranscript 26231	1352	2.75E-141	XP_002519102.1	1
Isopentenyl diphosphate delta isomerase	5.3.3.2	Unitranscript 3238, 53622, 53623,53624	1438, 881, 1539,1769	4.40E-159, 2.54E-129, 2.32E-159, 4.04E-140	XP_002514848.1	4
Squalene synthase	2.5.1.21	Unitranscript 658, 20308, 20313	2021, 1773, 1856	0,0,0	NP_001236365.1	3
Squalene monooxygenase	1.14.13.132	Unitranscript 81899	338	1.36E-068	XP_002530610.1	1
Polyneuridine-aldehyde esterase	3.1.1.78	Unitranscript 869,73905, 73906	1595, 1624, 1660	6.04E-135, 0, 1.99E-172	XP_002522352.1 XP_002510769.1	3
Strictosidine synthase	4.3.3.2	Unitranscript 53363, 53366, 62407, 62408	243, 1726, 1523, 831	1.01E-023, 0, 0, 3.97E-117	XP_002513740.1	4
Deacetoxyvindoline 4-hydroxylase	1.14.11.20	Unitranscript 3405, 3406, 17317,17320, 41143, 41144, 48984, 48986, 48987, 55158, 57629, 57630, 57631, 63714, 63716, 79369, 79370, 80577, 84582	1758, 1086, 1475, 1627, 2334,2415, 2336, 1508, 2179, 256, 1736, 1714, 531, 587, 717, 564, 664, 409, 337	0, 8.92E-128, 1.95E-177,0, 2.04E-096, 2.17E-095, 4.54E-171, 0, 3.73E-091, 2.49E-026, 9.26E-145, 0,1.24E-039, 1.38E-069, 4.43E-068, 1.31E-091, 9.17E-101, 3.40E-058, 1.44859E-11	XP_002529304.1 XP_002530339.1 XP_002525989.1 XP_002532376.1 XP_002529299.1	19
Polyphenol oxidase	1.10.3.1	Unitranscript 16967, 16969 16970, 16972, 16975	756, 952, 812, 813,478	1.43E-015, 9.29E-015, 5.75E-015, 5.75E-015, 7.89939E-13	XP_002316632.1	5
Amine oxidase	1.4.3.21	Unitranscript 63789,63790, 65487, 79801, 81672	692, 2340, 3132, 1096, 236	1.53E-075, 0, 0, 3.70E-173, 2.30E-034	XP_002509596.1 XP_002511334.1 XP_002278244.1 XP_002516781.1	5
N-methylcoclaurine 3′-monooxygenase	1.14.13.71	Unitranscript 77543, 79547	944,514	2.61E-102, 1.39E-067	XP_002510830.1	2
Reticuline oxidase	1.21.3.3	Unitranscript 3212, 70115, 70116, 79672	1094, 1725 1853, 802	2.91E-173, 0,0, 5.41E-156	XP_002523151.1 XP_002523157.1 XP_002523164.1	4

### Discovery of transcripts encoding transcription factors and their domain architecture

**Figure 10:**
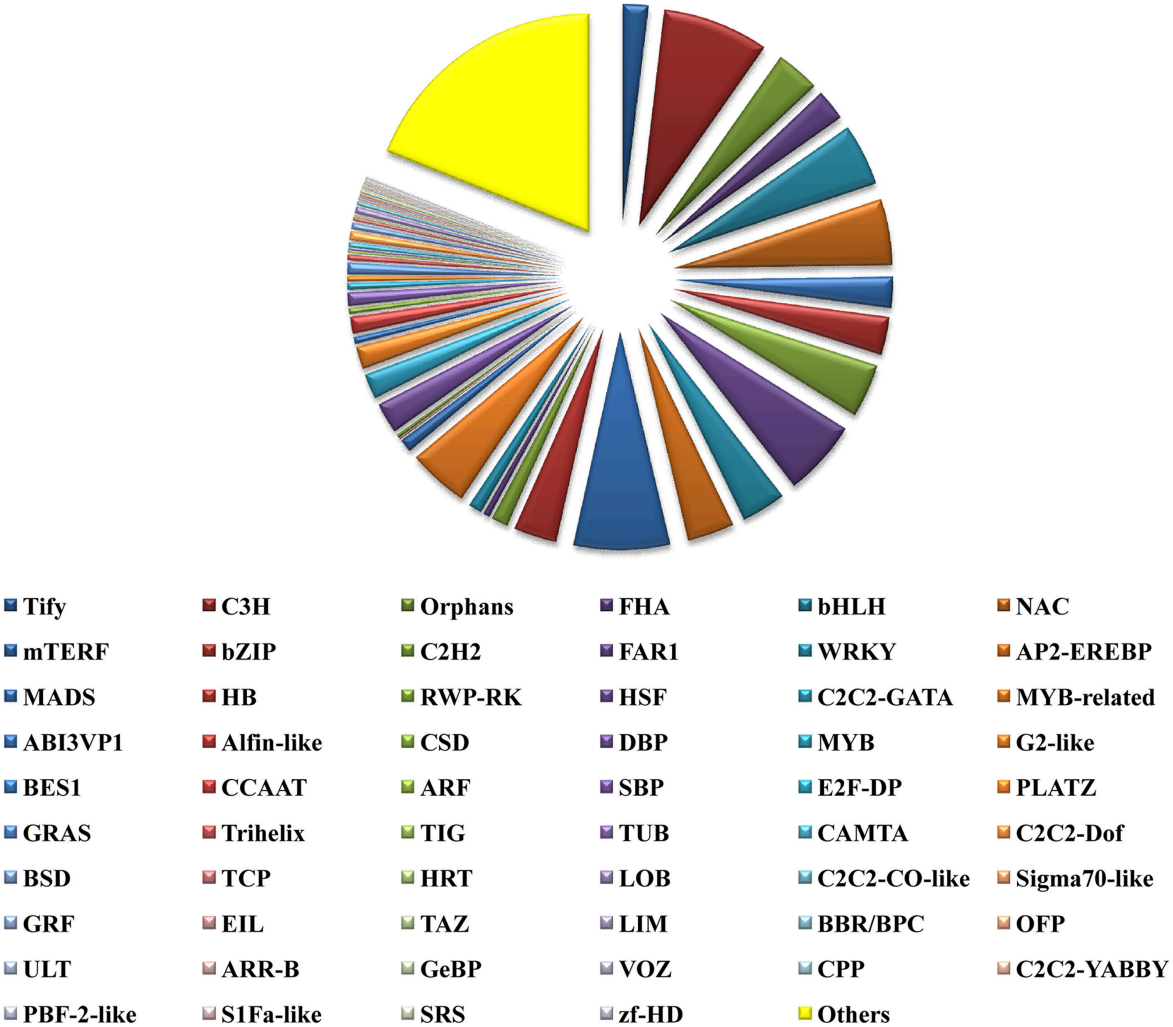
**Transcription factors identified from leaf transcriptome of *P. amarus de novo* assembled unitranscripts**.

TFs play key roles in controlling gene expression. In plants, TFs have been employed to manipulate various types of metabolic, developmental and stress response pathways. Further TFs are also known to regulate secondary metabolism in plants at the gene and protein levels as well (Vom Endt et al., 2002). TFs that are known to regulate plant secondary metabolism include R2R3-MYB, basic helix-loop-helix (bHLH) proteins like CrMYC2, AP2/ERF family proteins, WRKY, NAC, DOF, bZIP, HD-ZIP, and TFIIIA zinc finger TFs (Bhattacharyya et al., 2013). A total of 16,344 *P. amarus* unitranscripts could be annotated at the Plant TF database (PlnTFDB; http://plntfdb.bio.uni-potsdam.de/v3.0/downloads.php) (Pérez-Rodríguez et al., 2009) and categorized into 59 TF categories (Figure 10). The unitranscripts with their detailed TF protein identities and their corresponding domain annotations have been shown in Supplementary File 5. Among the annotated unitranscripts, notable unitranscripts identified and related to secondary metabolism were AP2-EREBP, NAC, bHLH, MYB, or MYB related, bZIP, mTERF, WRKY, zf-HD, C2C2-CO-like, and C2C2- Dof. Similar results were also obtained in our previous study of *de novo* transcriptome assembly of endangered medicinal herb *Podophyllum hexandrum* Royle whose extract podophyllotoxin is used for production of anticancer drugs (Bhattacharyya et al., 2013). Our present study has shown that 303 and 737 unitranscripts have encoded for MYB and MYB related TFs respectively. MYB TFs that regulate the phenylpropanoid biosynthetic pathway and also identified in several plant species, mostly include the R2R3-MYB TFs (Hichri et al., 2011; Bhattacharyya et al., 2013). Besides, R2R3-MYB TF has also been shown as a flavonol-specific regulator of phenylpropanoid biosynthesis in *Arabidopsis thaliana* (Mehrtens et al., 2005). Previous reports also show that in the grapevine phenylpropanoid pathway (Deluc et al., 2006) and its major branch viz. flavonoid biosynthetic pathway in *Prunus persica* and *Epimedium sagittatum* are both regulated by R2R3 MYB TFs (Huang et al., 2013; Ravaglia et al., 2013). Besides this, 750 unitranscripts coding for bHLH TFs have been identified in the present study. bHLH TFs like MYB also regulate the flavonoid biosynthetic pathway in plants. (Xu et al., 2015). Besides, we identified 1 Dof TF family in our present study. Dof TFs besides regulating diverse biological processes like carbon and nitrogen assimilation, dormancy, seed maturation and germination, phytochrome signaling, salicylic acid response, guard cell-specific gene expression, photoperiodic flowering, etc. have also been reported to influence phenylpropanoid metabolism in an environmental and tissue-specific manner (Skirycz et al., 2007; Gupta et al., 2015). Another TF family viz. WRKY has also been encoded by 541 unique sequences. WRKY proteins amongst its diverse functions are also involved in the biosynthesis of secondary metabolites (Eulgem et al., 2000). Taken together, TFs identified here can be evaluated further, especially those related to a wide array of secondary metabolites biosynthesis in this potential medicinal herb.

### *In silico* SSR mining and discovery

Microsatellites, or SSRs, are tandemly repeated short DNA sequence motifs ranging from 1 to 6 base pairs extensively distributed in eukaryotes, including the plants, animals and microorganisms, as well as in some prokaryotes (Morgante et al., 2002). SSRs have become one of the most widely used informative molecular markers because it is easy to detect and further used in several applications, including genetic diversity, evolution, genome mapping, marker assisted selection, and breeding studies. Out of 85,927 sequences that were examined by MISA tool, a total of 65,273 SSRs was identified out of which 29,652 were present in compound formation. Of the examined sequences 28,304 contained SSRs with 42% harboring more than one SSR. Statistical analysis of SSRs identified in our study has been presented in Table 3.

**Table 3:** **Statistics of SSRs identified from *P. amarus* leaf transcriptome**.

Total number of sequences examined	85,927
Total size of examined sequences (bp)	133,023,042
Total number of identified SSRs	65,273
Number of SSRs containing sequences	28,304
Number of sequences containing more than 1 SSR	11906
Number of SSRs present in compound formation	29,652

#### Frequency and distribution of different SSR repeat motifs

**Figure 11:**
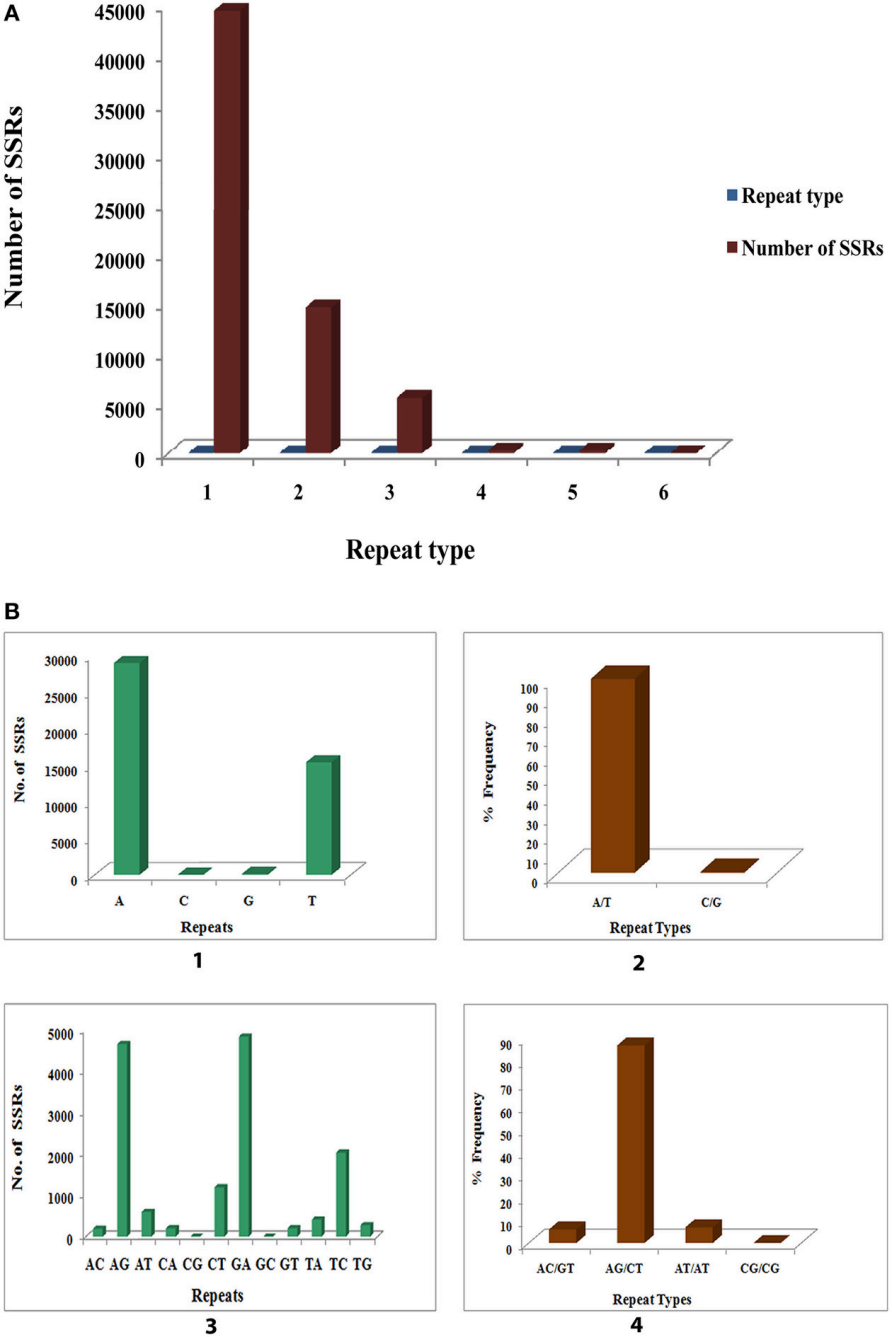
**Identification of molecular markers (SSRs) in leaf transcriptome of *P. amarus*. (A)** Distribution of SSR's into mono, di, tri, tetra, penta, and hexa repeat types. **(B)** Distribution of mono and di-nucleotide SSR motifs and percent frequency of their repeat types.

A summary of SSRs, including distribution of different repeat type classes, frequency of identified SSR motifs, and frequency of classified repeat types (considering sequence complementary) are shown in Supplementary File 6. Among each of the SSR classes the different possible repeat motifs were not evenly distributed. Among the identified SSRs mono-nucleotide repeat motif was the most abundant (44526, 68.215%) followed by di-nucleotide (14659, 22.458%), tri- nucleotide (5585, 8.556%), tetra-nucleotide (251, 0.385%), penta-nucleotide (236, 0.362%) and hexa-nucleotide (16, 0.025%) (Figure 11A). Of the mono-nucleotide repeats, adenine was the most abundant (65.06%) followed by thymine (34.564%), guanine (0.317%), and cytosine (0.061%) (Figure 11B1). The highest frequency observed for each of the identified mononucleotide SSR motif was 10 [(A)_10_, (T)_10_, (G)_10_ and (C)_10_]. Of the two possible types of mono-nucleotide repeats (considering sequence complementary), the most abundant was (A/T), as in most plants accounting for 99.623% as compared to the (C/G) type (Figure 11B2). The frequency for the different number of repeats of the (A/T) and (C/G) repeat types as shown was maximum for (A/T)_10_ and (C/G)_10_ accounting for 20.5 and 26.19% respectively (Figure 11B2). The dinucleotide repeat (GA)_12_ was the most abundant (33.079%) (Figure 11B3) with the repeat type (AG/CT)_12_ having the maximum frequency (Figure 11B4). With respect to tri- nucleotide repeats (GAA)_8_ was the most abundant (13.823%) with the repeat type (AAG/CTT)_5_ having the highest frequency (36.115%) (Supplementary File 6). The most frequent tetra- nucleotide repeat motif was (AGAA)_6_ (9.562%). (AAAG/CTTT)_5_ was the most abundant tetra-nucleotide repeat type occurring 28.287%. (Supplementary File 6). Among the penta- and hexa-nucleotide repeat motifs the most abundant were (TCTCT)_5_ (12.288%) and (AAGCCA)_6_ (68.75%) respectively (Supplementary File 6). (AAGAG/CTCTT)_5_ (20.763%) and (AAAGCC/CTTTGG)_5_ (68.75%) were the repeat types with highest frequency among the penta- and hexa-nucleotide repeat motifs respectively (Supplementary File 6). The details of SSR types obtained for the *P. amarus* unitranscripts have been shown in Supplementary File 7.

The large number of SSRs thus identified for the first time from the leaf transcriptome of this medicinal herb will facilitate gene mapping, secondary metabolite improvement, and enable genetic diversity analysis in future on *P. amarus* genomics study.

### RT-PCR for validation and FPKM value determination of the major secondary metabolite pathway genes from *P. amarus* leaf

The *de novo* assembled and annotated unitranscripts of *P. amarus* leaf transcriptome were further validated. We selected some of the unitranscripts annotated against the multiple databases that revealed putative information of *P. amarus* leaf transcriptome. The FPKM values of the major secondary metabolite pathway genes that were identified from the *P. amarus* leaf transcriptome as mentioned in Tables 1, 2 were also calculated to further correlate the putative unitranscript abundance with their expression in the leaf tissues of this herb (Supplementary File 8). Some of the significantly expressed unitranscripts with FPKM values on the higher side, identified as the major secondary metabolic pathway genes in *P. amarus* leaf transcriptome included—phenylalanine ammonia lyase (PAL), cinnamate 4-hydroxylase/trans-cinnamate 4-monooxygenase, flavonoid 3′,5′-hydroxylase, phenylcoumaran benzylic ether reductase -like protein, HMG-CoA synthase, mevalonate diphosphate decarboxylase, isopentenyl diphosphate delta isomerase, strictosidine synthase, deacetoxyvindoline 4-hydroxylase, 1-deoxy-D-xylulose-5-phosphate reductoisomerase, polyphenol oxidase, amine oxidase, squalene synthase, chalcone isomerase (Supplementary File 8). Based on the putative findings and correlating with the FPKM values, a few of the secondary metabolic genes were selected for performing Reverse Transcription PCR (RT-PCR) to validate the reliability and accuracy of our *de novo* assembled *P. amarus* leaf transcriptome data.

We identified 7 of the selected genes while performing RT-PCR with the leaf sample, each from the lignans biosynthetic pathway genes like phenylcoumaran benzylic ether reductase -like protein (Unitranscript 63241; GO:0009807: lignan biosynthetic process); phenylcoumaran benzylic ether reductase -like protein (Unitranscript 77577; GO:0010283: pinoresinol reductase activity); and each from phenylpropanoid, flavonoid, and alkaloid biosynthetic pathways like phenylalanine ammonia lyase, putative (Unitranscript 31404; GO:0045548: phenylalanine ammonia lyase activity); chalcone isomerase (Unitranscript 5340; GO:0009813: flavonoid biosynthetic process, GO:0045430: chalcone isomerase activity); strictosidine synthase, putative (Unitranscript 53363; GO:0016844: strictosidine synthase activity) respectively. Also, two TFs (related to the secondary metabolites biosynthesis) like R2R3-MYB TF (Unitranscript 1528) and bHLH TF (Unitranscript 14718) were selected for RT-PCR validation.

**Figure 12:**
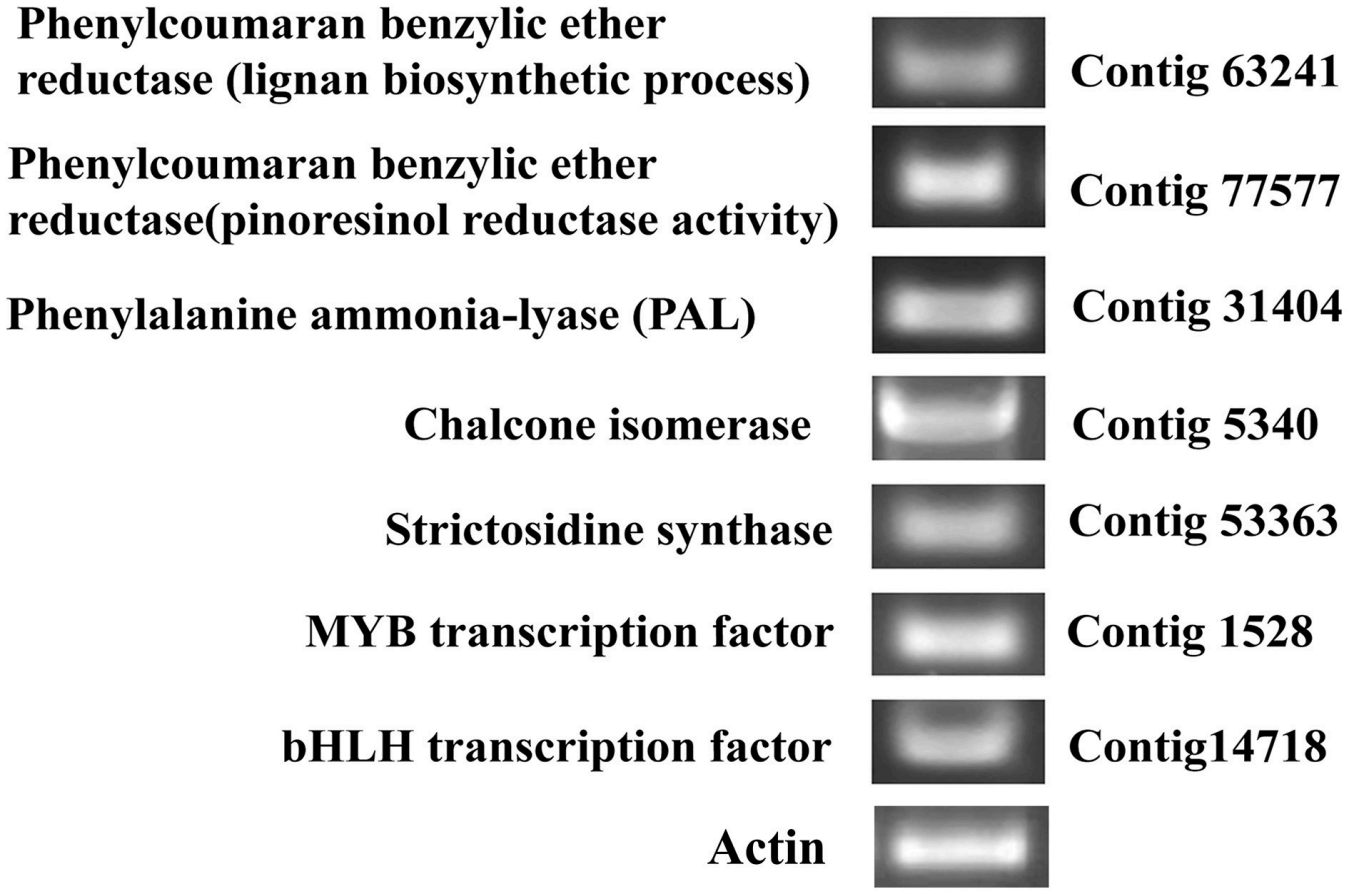
**RT-PCR image of selected *P. amarus* unitranscripts expressed in leaf samples of *P. amarus***.

Experimentally confirmed data of gene expression provide a preferable understanding of the function and regulation of the genes. Hence, we performed RT-PCR of the above mentioned unitranscripts (Figure 12) identified in our putative leaf transcriptome dataset to confirm the gene expression of these unitranscripts along with *actin* gene used as a control in the leaf tissue samples of *P. amarus*. Our results suggested that the three-level *de novo* assembly and annotation data of our present study can be used in genomics study and practical experiments on *P. amarus* in future.

## Conclusion

*P. amarus* transcriptome research lags that of other plants of medicinal importance. To facilitate molecular research in *P. amarus* we characterized the leaf transcriptome since the vast repertoire of secondary metabolites are mainly present in the leaf tissues of this herb. Our data on leaf transcriptome analysis using the Illumina MiSeq platform have instigated to the identification of a large number of transcripts, TFs, molecular/SSR markers involved in diverse processes, functions, metabolic pathways together with the transcripts involved in a number of secondary metabolic pathways, specially attributing to the phytomedicinal significance of this herb. To be more precise, the putative transcriptome information explored in our data revealing the various lignans, flavonoids, terpenoids, alkaloids and other secondary metabolites biosynthesis pathway genes adds a copious amount of information to *P. amarus* database and also paves the way for functional and comparative genomic studies of this highly promising medicinal plant in future. Further RT-PCR results showing expression of the few selected unitranscripts, involved in various classes of secondary metabolites synthesis, also confirmed the reliability and accuracy of our *P. amarus* leaf transcriptome assembly. This is the first report on a detailed study of *P. amarus* leaf transcriptome that was done to provide an important resource for future studies on “bhuiamalaki” thereby greatly facilitating research on non-model plants in plant biology.

## Author contributions

AB and SC designed the experiment. AB carried out the experimental work, analyzed the data and drafted the manuscript. SC supervised the analysis of NGS data and prepared the final manuscript.

### Conflict of interest statement

The authors declare that the research was conducted in the absence of any commercial or financial relationships that could be construed as a potential conflict of interest.

## Supplementary material

Supplementary Figure 1 **Summary of filtration report of total raw reads generated and used for transcriptome assembly**.

Supplementary Figure 2 **Terpenoid biosynthesis pathway**.

Supplementary Figure 3 **Isoquinoline alkaloid biosynthesis pathway**.

Supplementary Figure 4 **Tropane, piperidine, and pyridine alkaloid biosynthetic pathway genes identified**.

Supplementary Figure 5 **Putatively identified genes of diterpenoid biosynthesis pathway**.
