# Supplementary figures and images for: Sequencing, De novo Assembly, Functional Annotation and Analysis of Phyllanthus amarus Leaf Transcriptome Using the Illumina Platform
Source: Front Plant Sci. 2016 Jan 28;6:1199. doi: 10.3389/fpls.2015.01199 (PMC4729934; doi:10.3389/fpls.2015.01199)

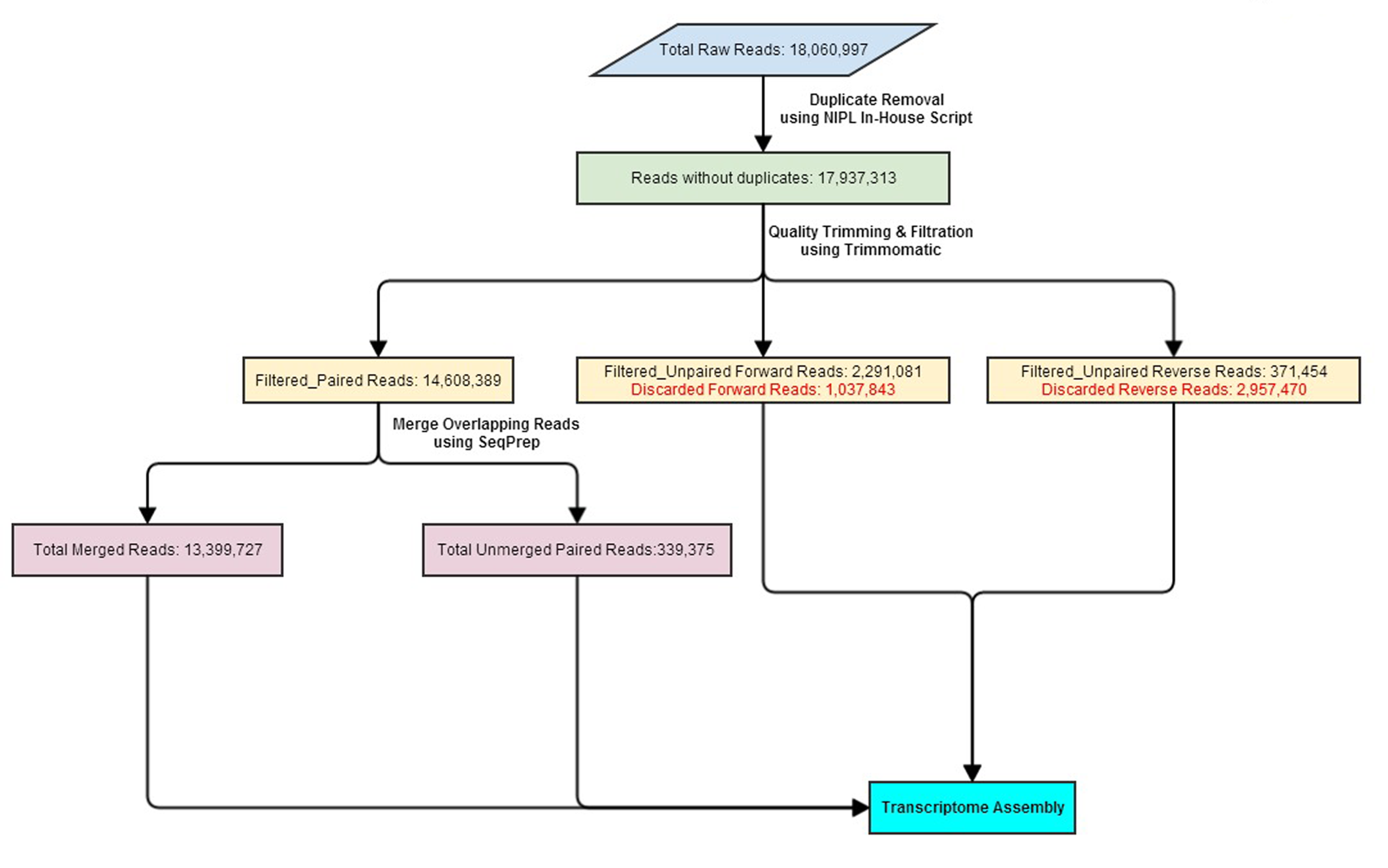

Supplement: Supplementary Figure 1 — Summary of filtration report of total raw reads generated and used for transcriptome assembly. [file Image1.TIF]

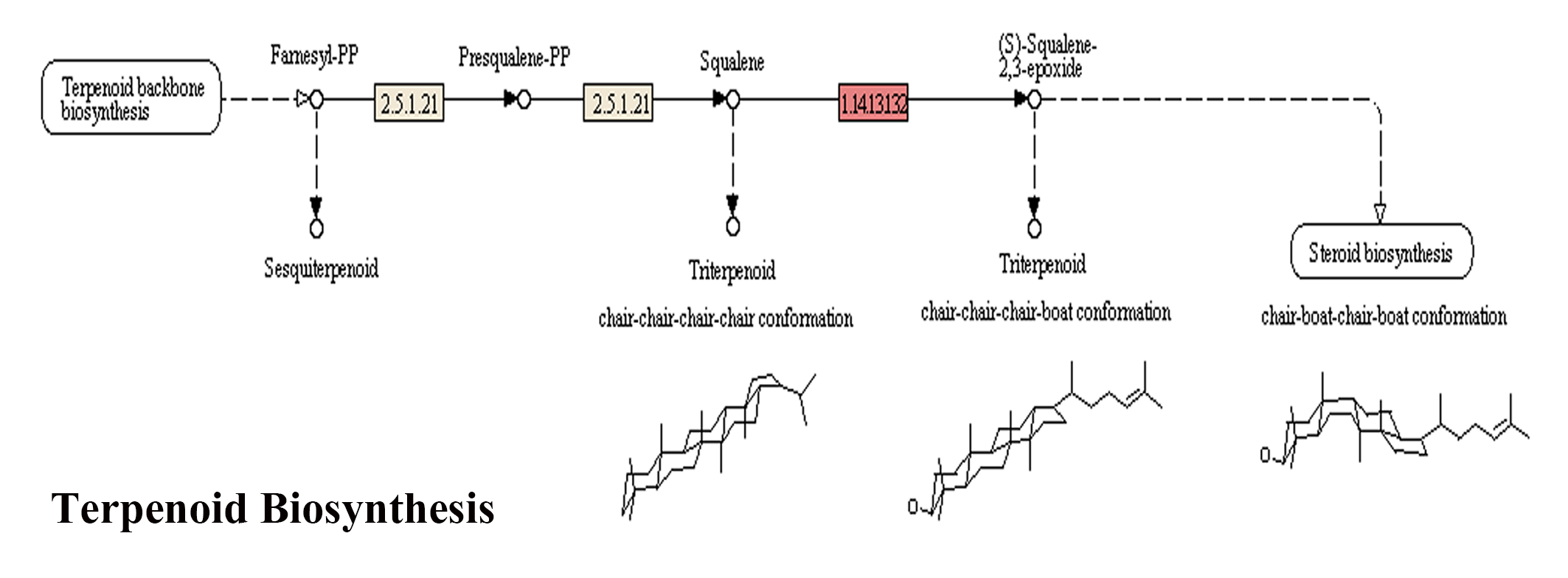

Supplement: Supplementary Figure 2 — Terpenoid biosynthesis pathway. [file Image2.tif]

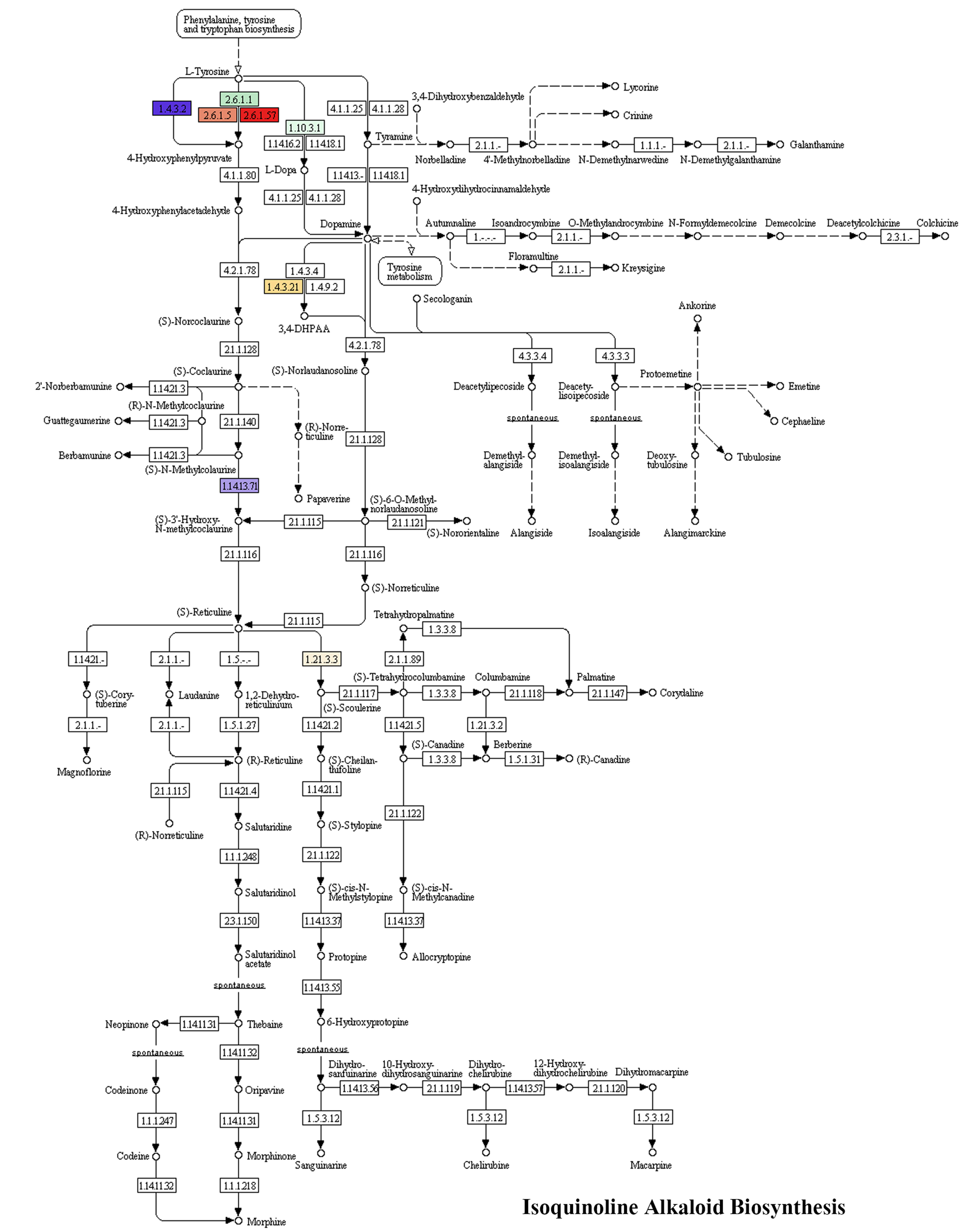

Supplement: Supplementary Figure 3 — Isoquinoline alkaloid biosynthesis pathway. [file Image3.TIF]

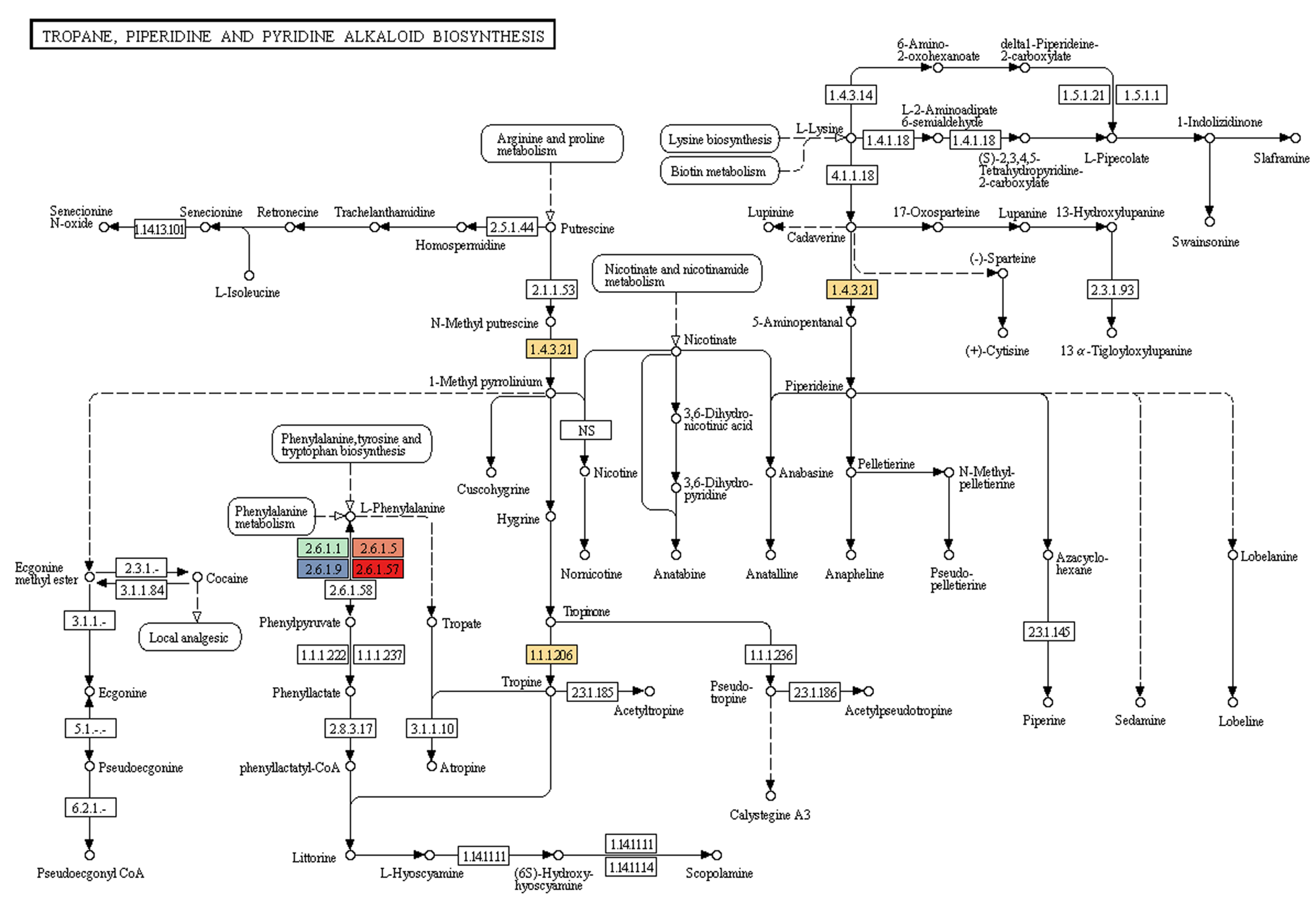

Supplement: Supplementary Figure 4 — Tropane, piperidine, and pyridine alkaloid biosynthetic pathway genes identified. [file Image4.TIF]

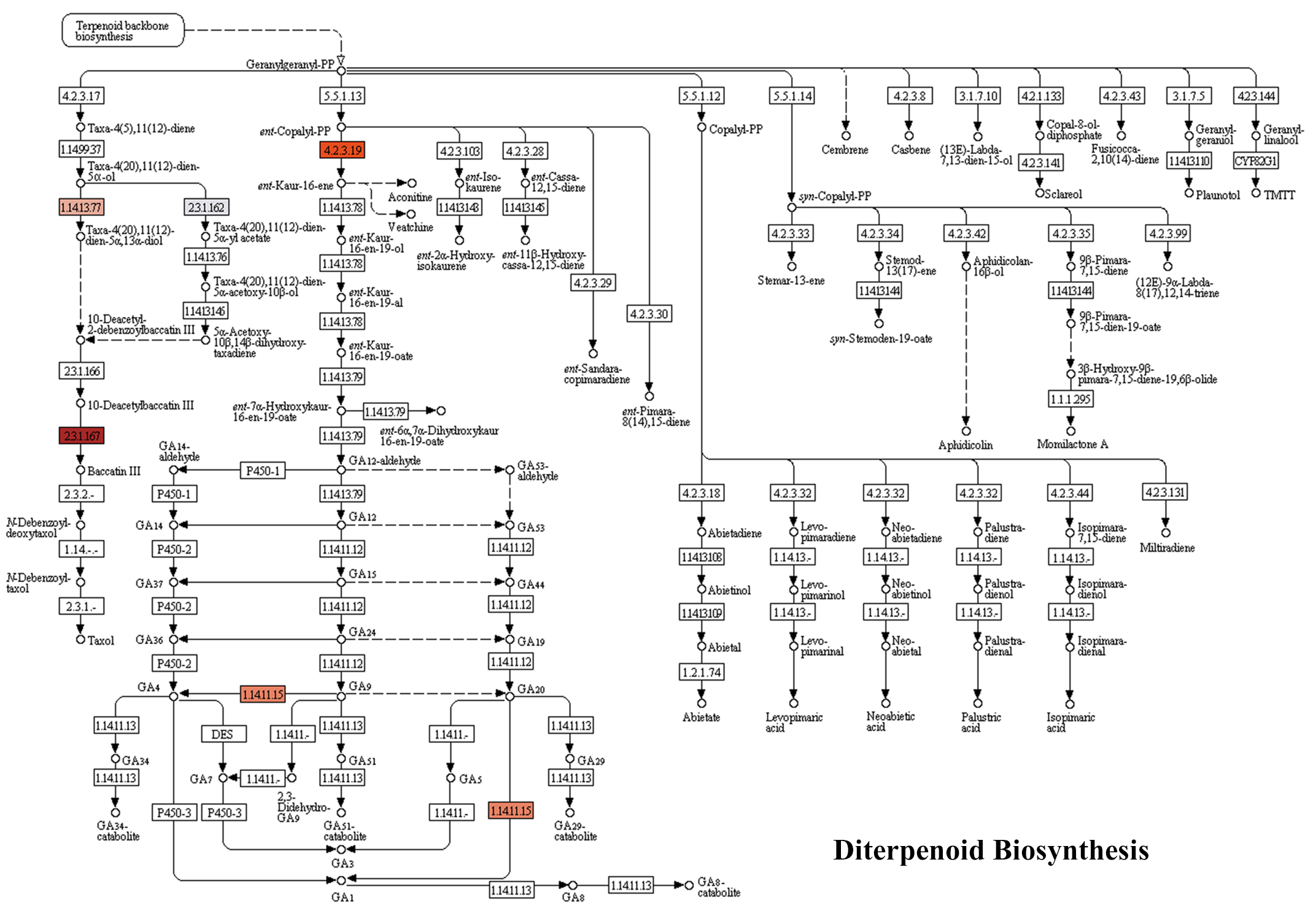

Supplement: Supplementary Figure 5 — Putatively identified genes of diterpenoid biosynthesis pathway. [file Image5.TIF]
