# Supplementary material for: Sequencing, De novo Assembly, Functional Annotation and Analysis of Phyllanthus amarus Leaf Transcriptome Using the Illumina Platform
Source: Front Plant Sci. 2016 Jan 28;6:1199. doi: 10.3389/fpls.2015.01199 (PMC4729934; doi:10.3389/fpls.2015.01199)
Supplement: Supplementary file 6 [file Table1.DOC]

**Supplementary Table S1: Level 1 assembly parameters of the *P. amarus* filtered sequence data using Velvet & Oases.**

| **Kmer** | **No. of Transcripts** | **Total length** | **Average size** | **N50** | **Maximum transcript size** | **Minimum transcript size** |
| --- | --- | --- | --- | --- | --- | --- |
| 27 | 75530 | 117759458 | 1559.1084072554 | 2294 | 11628 | 100 |
| 29 | 76875 | 118964859 | 1547.51036097561 | 2286 | 10497 | 100 |
| 31 | 136427 | 102755198 | 753.18813724556 | 1850 | 10520 | 100 |
| 33 | 78791 | 124669451 | 1582.28034927847 | 2302 | 11593 | 100 |
| 35 | 78771 | 123148075 | 1563.36818118343 | 2294 | 11595 | 100 |
| 37 | 77849 | 123043455 | 1580.53995555498 | 2285 | 11697 | 100 |
| 39 | 137419 | 104782956 | 762.507047788152 | 1825 | 7592 | 100 |
| 41 | 76228 | 119627534 | 1569.33848454636 | 2263 | 11366 | 100 |
| 43 | 74732 | 116932778 | 1564.69488304876 | 2257 | 11368 | 100 |
| 45 | 94803 | 110579482 | 1166.41332025358 | 2104 | 11612 | 100 |
| 47 | 72381 | 111121929 | 1535.23616694989 | 2233 | 11614 | 100 |
| 49 | 69573 | 106525766 | 1531.13659034396 | 2211 | 11616 | 100 |
| 51 | 90215 | 101716426 | 1127.48906501136 | 2022 | 10933 | 100 |
| 53 | 66565 | 100763842 | 1513.76612333809 | 2189 | 11220 | 100 |
| 55 | 91917 | 97636996 | 1062.23001185852 | 2006 | 8187 | 100 |
| 57 | 93901 | 94035445 | 1001.43177388952 | 1957 | 8188 | 100 |
| 59 | 123335 | 85874553 | 696.270750395265 | 1586 | 7749 | 100 |
| 61 | 105368 | 85631348 | 812.688368385088 | 1775 | 8219 | 100 |
| 63 | 59270 | 83750417 | 1413.03217479332 | 2110 | 8202 | 100 |
