# Supplementary material for: Sequencing, De novo Assembly, Functional Annotation and Analysis of Phyllanthus amarus Leaf Transcriptome Using the Illumina Platform
Source: Front Plant Sci. 2016 Jan 28;6:1199. doi: 10.3389/fpls.2015.01199 (PMC4729934; doi:10.3389/fpls.2015.01199)
Supplement: Supplementary file 7 [file Table2.DOC]

**Supplementary Table S**2: Statistics of level 2 and level 3 assemblies of NGS reads using Velvet & Oases and CD-HIT softwares respectively.

|  | **Total**  **Number** | **Total length** | **Average transcript size** | **N50** | **Maximum Transcript size** | **Minimum Transcript size** |
| --- | --- | --- | --- | --- | --- | --- |
| **Transcript** | 3,60,405 | 574190085 | 1593.18013068631 | 2289 | 13,600 | 100 |
| **Contigs or “Unitranscript”** | 85,927 | 133023042 | 1548.09363762263 | 2182 | 13,600 | 200 |
