# Supplementary material for: Sequencing, De novo Assembly, Functional Annotation and Analysis of Phyllanthus amarus Leaf Transcriptome Using the Illumina Platform
Source: Front Plant Sci. 2016 Jan 28;6:1199. doi: 10.3389/fpls.2015.01199 (PMC4729934; doi:10.3389/fpls.2015.01199)
Supplement: Supplementary file 8 [file Table3.DOC]

**Supplementary Table S3. Annotation summary of *P. amarus* unitranscripts in different databases.**

| **Database** | **Hits** |
| --- | --- |
| Green plants non-redundant Nr protein | 70,662 |
| Gene Ontology (GO) | 20,582 |
| The Cluster of Orthologous Groups (COG) | 28,121 |
| KEGG | 4,697 |
| PlnTFDB | 16,344 |
