# Supplementary material for: Sequencing, De novo Assembly, Functional Annotation and Analysis of Phyllanthus amarus Leaf Transcriptome Using the Illumina Platform
Source: Front Plant Sci. 2016 Jan 28;6:1199. doi: 10.3389/fpls.2015.01199 (PMC4729934; doi:10.3389/fpls.2015.01199)
Supplement: Supplementary file 9 [file Table4.DOC]

**Supplementary Table S4. Comparison of *P. amarus* assembled leaf transcriptome with closely sequenced species using TRAPID Analysis**

1. **Comparison with *Populus trichocarpa***

| **Transcript Information** | | | |
| --- | --- | --- | --- |
| Unitranscripts | | | 85927 |
| Average unitranscript length | | | 1548.1 bp |
| **Meta Annotation Information** | | | |
| Meta annotation full-length | | 34950 (40.7%) | |
| Meta annotation quasi full-length | | 13820 (16.1%) | |
| Meta annotation partial | | 22347 (26%) | |
| Meta annotation no information | | 14810 (17.2%) | |
| **Similarity Search Information** | | | |
| *Populus trichocarpa* | 71896 (83.7%) | | |
| **Gene Family Information** | | | |
| Gene families | | | 6410 |
| Unitranscripts in GF | | | 71896 (83.7%) |
| **Functional Annotation Information** | | | |
| Unitranscripts with GO | | | 53478 (62.2%) |
| Unitranscripts with Protein Domain | | | 61971 (72.1%) |

1. **Comparison with *Ricinus communis***

| **Transcript information** | | | |
| --- | --- | --- | --- |
| Unitranscripts | | 85927 | |
| Average unitranscript length | | 1548.1 bp | |
| **Meta annotation information** | | | |
| Meta annotation full-length | 34737 (40.4%) | | |
| Meta annotation quasi full-length | 13835 (16.1%) | | |
| Meta annotation partial | 22392 (26.1%) | | |
| Meta annotation no information | 14963 (17.4%) | | |
| **Similarity Search Information** | | | |
| *Ricinus communis* | | | 71358 (83%) |
| **Gene Family Information** | | | |
| Gene families | | | 6164 |
| Unitranscripts in GF | | | 71358 (83%) |
| **Functional Annotation Information** | | | |
| Unitranscripts with GO | | | 53226 (61.9%) |
| Unitranscripts with Protein Domain | | | 61652 (71.7%) |
