# Supplementary material for: Sequencing, De novo Assembly, Functional Annotation and Analysis of Phyllanthus amarus Leaf Transcriptome Using the Illumina Platform
Source: Front Plant Sci. 2016 Jan 28;6:1199. doi: 10.3389/fpls.2015.01199 (PMC4729934; doi:10.3389/fpls.2015.01199)
Supplement: Supplementary file 10 [file Table5.DOC]

**Supplementary Table S5. Annotation and classification of *P. amarus* unitranscripts into secondary metabolic pathways.**

| **Secondary Metabolism Pathways** | **Number of Unitranscripts** |
| --- | --- |
| Terpenoid backbone biosynthesis | 72 |
| Monoterpenoid biosynthesis | 18 |
| Sesquiterpenoid and triterpenoid biosynthesis | 4 |
| Diterpenoid biosynthesis | 25 |
| Carotenoid biosynthesis | 10 |
| Zeatin biosynthesis | 27 |
| Limonene and pinene degradation | 28 |
| Geraniol degradation | 22 |
| Biosynthesis of ansamycins | 7 |
| Tetracycline biosynthesis | 3 |
| Polyketide sugar unit biosynthesis | 1 |
| Biosynthesis of siderophore group nonribosomal peptides | 1 |
| Phenylpropanoid biosynthesis | 125 |
| Stilbenoid, diarylheptanoid and gingerol biosynthesis | 4 |
| Flavonoid biosynthesis | 134 |
| Flavone and flavonol biosynthesis | 34 |
| Anthocyanin biosynthesis | 37 |
| Isoflavonoid biosynthesis | 5 |
| Indole alkaloid biosynthesis | 28 |
| Isoquinoline alkaloid biosynthesis | 51 |
| Tropane, piperidine and pyridine alkaloid biosynthesis | 65 |
| Caffeine metabolism | 26 |
| Glucosinolate biosynthesis | 22 |
| Penicillin and cephalosporin biosynthesis | 4 |
| Streptomycin biosynthesis | 22 |
| Butirosin and neomycin biosynthesis | 3 |
| Novobiocin biosynthesis | 41 |
| Aflatoxin biosynthesis | 3 |
| Benzoate degradation | 48 |
| Aminobenzoate degradation | 89 |
| Fluorobenzoate degradation | 19 |
| Chloroalkane and chloroalkene degradation | 23 |
| Chlorocyclohexane and chlorobenzene degradation | 21 |
| Toluene degradation | 33 |
| Ethylbenzene degradation | 4 |
| Styrene degradation | 49 |
| Atrazine degradation | 3 |
| Caprolactam degradation | 12 |
| Naphthalene degradation | 5 |
| Steroid degradation | 13 |
| Metabolism of xenobiotics by cytochrome P450 | 67 |
| Drug metabolism - cytochrome P450 | 68 |
| Drug metabolism - other enzymes | 56 |
