# Supplementary material for: Sequencing, De novo Assembly, Functional Annotation and Analysis of Phyllanthus amarus Leaf Transcriptome Using the Illumina Platform
Source: Front Plant Sci. 2016 Jan 28;6:1199. doi: 10.3389/fpls.2015.01199 (PMC4729934; doi:10.3389/fpls.2015.01199)
Supplement: Supplementary file 11 [file Table6.DOC]

**Supplementary Table S6. KEGG pathway categorization of assembled *P. amarus* leaf transcriptome unitranscripts**

| **1.Metabolism** | | | | |
| --- | --- | --- | --- | --- |
| **1.1 Carbohydrate metabolism** | | **Number of Unitranscripts** | | **Pathway ID** |
| Glycolysis / Gluconeogenesis | | 224 | | map00010 |
| Citrate cycle (TCA cycle) | | 143 | | map00020 |
| Pentose phosphate pathway | | 179 | | map00030 |
| Pentose and glucuronate interconversions | | 134 | | map00040 |
| Fructose and mannose metabolism | | 119 | | map00051 |
| Galactose metabolism | | 216 | | map00052 |
| Ascorbate and aldarate metabolism | | 61 | | map00053 |
| Starch and sucrose metabolism | | 412 | | map00500 |
| Amino sugar and nucleotide sugar metabolism | | 208 | | map00520 |
| Pyruvate metabolism | | 245 | | map00620 |
| Glyoxylate and dicarboxylate metabolism | | 167 | | map00630 |
| Propanoate metabolism | | 105 | | map00640 |
| Butanoate metabolism | | 104 | | map00650 |
| C5-Branched dibasic acid metabolism | | 23 | | map00660 |
| Inositol phosphate metabolism | | 84 | | map00562 |
| **1.2 Energy metabolism** | | | | |
| Oxidative phosphorylation | | 90 | | map00190 |
| Photosynthesis | | 6 | | map00195 |
| Carbon fixation in photosynthetic organisms | | 150 | | map00710 |
| Oxidative phosphorylation | | 90 | | map00190 |
| Carbon fixation pathways in prokaryotes | | 135 | | map00720 |
| Methane metabolism | | 138 | | map00680 |
| Nitrogen metabolism | | 65 | | map00910 |
| Sulfur metabolism | | 51 | | map00920 |
| **1.3 Lipid metabolism** | | | | |
| Fatty acid biosynthesis | | 52 | | map00061 |
| Fatty acid elongation | | 32 | | map00062 |
| Fatty acid degradation | | 144 | | map00071 |
| Synthesis and degradation of ketone bodies | | 41 | | map00072 |
| Cutin, suberine and wax biosynthesis | | 46 | | map00073 |
| Steroid biosynthesis | | 35 | | map00100 |
| Primary bile acid biosynthesis | | 7 | | map00120 |
| Steroid hormone biosynthesis | | 43 | | map00140 |
| Glycerolipid metabolism | | 205 | | map00561 |
| Glycerophospholipid metabolism | | 160 | | map00564 |
| Ether lipid metabolism | | 43 | | map00565 |
| Sphingolipid metabolism | | 84 | | map00600 |
| Arachidonic acid metabolism | | 63 | | map00590 |
| Linoleic acid metabolism | | 45 | | map00591 |
| alpha-Linolenic acid metabolism | | 75 | | map00592 |
| Biosynthesis of unsaturated fatty acids | | 68 | | map01040 |
| **1.4 Nucleotide metabolism** | | | | |
| Purine metabolism | | 467 | | map00230 |
| Pyrimidine metabolism | | 170 | | map00240 |
| **1.5 Amino acid metabolism** | | | | |
| Alanine, aspartate and glutamate metabolism | | 115 | | map00250 |
| Glycine, serine and threonine metabolism | | 147 | | map00260 |
| Cysteine and methionine metabolism | | 169 | | map00270 |
| Valine, leucine and isoleucine biosynthesis | | 75 | | map00290 |
| Valine, leucine and isoleucine degradation | | 175 | | map00280 |
| Lysine biosynthesis | | 86 | | map00300 |
| Lysine degradation | | 124 | | map00310 |
| Arginine and proline metabolism | | 205 | | map00330 |
| Histidine metabolism | | 52 | | map00340 |
| Tyrosine metabolism | | 124 | | map00350 |
| Phenylalanine metabolism | | 195 | | map00360 |
| Tryptophan metabolism | | 96 | | map00380 |
| Phenylalanine, tyrosine and tryptophan biosynthesis | | 177 | | map00400 |
| **1.6 Metabolism of other amino acids** | | | | |
| beta-Alanine metabolism | | 91 | | map00410 |
| Taurine and hypotaurine metabolism | | 50 | | map00430 |
| Phosphonate and phosphinate metabolism | | 10 | | map00440 |
| Selenocompound metabolism | | 38 | | map00450 |
| Cyanoamino acid metabolism | | 73 | | map00460 |
| D-Arginine and D-ornithine metabolism | | 8 | | map00472 |
| D-Alanine metabolism | | 8 | | map00473 |
| Glutathione metabolism | | 173 | | map00480 |
| **1.7 Glycan biosynthesis and metabolism** | | | | |
| N-glycan biosynthesis | | 33 | | map00510 |
| Various types of N-glycan biosynthesis | | 14 | | map00513 |
| Other types of O-glycan biosynthesis | | 16 | | map00514 |
| Glycosaminoglycan biosynthesis - chondroitin sulfate / dermatan sulfate | | 51 | | map00532 |
| Glycosaminoglycan biosynthesis - heparan sulfate / heparin | | 61 | | map00534 |
| Glycosaminoglycan degradation | | 39 | | map00531 |
| Glycosylphosphatidylinositol(GPI)-anchor biosynthesis | | 1 | | map00563 |
| Glycosphingolipid biosynthesis - lacto and neolacto series | | 1 | | map00601 |
| Glycosphingolipid biosynthesis - globo series | | 21 | | map00603 |
| Glycosphingolipid biosynthesis - ganglio series | | 35 | | map00604 |
| Lipopolysaccharide biosynthesis | | 7 | | map00540 |
| Peptidoglycan biosynthesis | | 6 | | map00550 |
| Other glycan degradation | | 47 | | map00511 |
| **1.8 Metabolism of cofactors and vitamins** | | | | |
| Thiamine metabolism | | 159 | | map00730 |
| Riboflavin metabolism | | 50 | | map00740 |
| Vitamin B6 metabolism | | 10 | | map00750 |
| Nicotinate and nicotinamide metabolism | | 50 | | map00760 |
| Pantothenate and CoA biosynthesis | | 59 | | map00770 |
| Biotin metabolism | | 21 | | map00780 |
| Lipoic acid metabolism | | 1 | | map00785 |
| Folate biosynthesis | | 10 | | map00790 |
| One carbon pool by folate | | 38 | | map00670 |
| Retinol metabolism | | 41 | | map00830 |
| Porphyrin and chlorophyll metabolism | | 100 | | map00860 |
| Ubiquinone and other terpenoid-quinone biosynthesis | | 90 | | map00130 |
| **1.9 Metabolism of terpenoids and polyketides** | | | | |
| Terpenoid backbone biosynthesis | | 72 | | map00900 |
| Monoterpenoid biosynthesis | | 18 | | map00902 |
| Sesquiterpenoid and triterpenoid biosynthesis | | 4 | | map00909 |
| Diterpenoid biosynthesis | | 25 | | map00904 |
| Carotenoid biosynthesis | | 10 | | map00906 |
| Zeatin biosynthesis | | 27 | | map00908 |
| Limonene and pinene degradation | | 28 | | map00903 |
| Geraniol degradation | | 22 | | map00281 |
| Biosynthesis of ansamycins | | 7 | | map01051 |
| Tetracycline biosynthesis | | 3 | | map00253 |
| Polyketide sugar unit biosynthesis | | 1 | | map00523 |
| Biosynthesis of siderophore group nonribosomal peptides | | 1 | | map01053 |
| **1.10 Biosynthesis of other secondary metabolites** | | | | |
| Phenylpropanoid biosynthesis | | 125 | | map00940 |
| Stilbenoid, diarylheptanoid and gingerol biosynthesis | | 4 | | map00945 |
| Flavonoid biosynthesis | | 134 | | map00941 |
| Flavone and flavonol biosynthesis | | 34 | | map00944 |
| Anthocyanin biosynthesis | | 37 | | map00942 |
| Isoflavonoid biosynthesis | | 5 | | map00943 |
| Indole alkaloid biosynthesis | | 28 | | map00901 |
| Isoquinoline alkaloid biosynthesis | | 51 | | map00950 |
| Tropane, piperidine and pyridine alkaloid biosynthesis | | 65 | | map00960 |
| Caffeine metabolism | | 26 | | map00232 |
| Glucosinolate biosynthesis | | 22 | | map00966 |
| Penicillin and cephalosporin biosynthesis | | 4 | | map00311 |
| Streptomycin biosynthesis | | 22 | | map00521 |
| Butirosin and neomycin biosynthesis | | 3 | | map00524 |
| Novobiocin biosynthesis | | 41 | | map00401 |
| Aflatoxin biosynthesis | | 3 | | map00254 |
| **1.11 Xenobiotics biodegradation and metabolism** | | | | |
| Benzoate degradation | 48 | | map00362 | |
| Aminobenzoate degradation | 89 | | map00627 | |
| Fluorobenzoate degradation | 19 | | map00364 | |
| Chloroalkane and chloroalkene degradation | 23 | | map00625 | |
| Chlorocyclohexane and chlorobenzene degradation | 21 | | map00361 | |
| Toluene degradation | 33 | | map00623 | |
| Ethylbenzene degradation | 4 | | map00642 | |
| Styrene degradation | 49 | | map00643 | |
| Atrazine degradation | 3 | | map00791 | |
| Caprolactam degradation | 12 | | map00930 | |
| Naphthalene degradation | 5 | | map00626 | |
| Steroid degradation | 13 | | map00984 | |
| Metabolism of xenobiotics by cytochrome P450 | 67 | | map00980 | |
| Drug metabolism - cytochrome P450 | 68 | | map00982 | |
| Drug metabolism - other enzymes | 56 | | map00983 | |
| **2. Genetic Information Processing** | | | | |
| **Translation** | | | | |
| Aminoacyl-tRNA biosynthesis | 78 | | map00970 | |
| **3. Environmental Information Processing** | | | | |
| **Signal transduction** | | | | |
| Phosphatidylinositol signaling system | 146 | | map04070 | |
| mTOR signaling pathway | 45 | | map04150 | |
| **4. Organismal Systems** | | | | |
| **Immune system** | | | | |
| T cell receptor signaling pathway | 303 | | map04660 | |
| **5. Human Diseases** | | | | |
| **Drug resistance** | | | | |
| beta-Lactam resistance | 2 | | map00312 | |
